# Transformation of normal cells by aberrant activation of YAP via cMyc with TEAD

**DOI:** 10.1038/s41598-019-47301-6

**Published:** 2019-07-29

**Authors:** Masazumi Nishimoto, Kousuke Uranishi, Masamitsu N. Asaka, Ayumu Suzuki, Yosuke Mizuno, Masataka Hirasaki, Akihiko Okuda

**Affiliations:** 1 Division of Developmental Biology, Research Center for Genomic Medicine, Saitama Medical University, 1397-1 Yamane Hidaka, Saitama, 350-1241 Japan; 2 Division of Radioisotope, Saitama Medical University, 1397-1 Yamane Hidaka, Saitama, 350-1241 Japan; 3 Division of Analytical Science, Hidaka Branch, Biomedical Research Center, Saitama Medical University, 1397-1 Yamane Hidaka, Saitama, 350-1241 Japan

**Keywords:** Cancer, HIPPO signalling

## Abstract

YAP (also known as YAP1 or YAP65) is a transcriptional coactivator that interacts with a number of transcription factors including RUNX and TEAD and plays a pivotal role in controlling cell growth. *YAP* is classified as a proto-oncogene. However, the mechanism by which activated YAP induces cancerous changes is not well known. Here we demonstrate that overexpression of YAP in NIH3T3 cells was sufficient for inducing tumorigenic transformation of cells. Mechanistically, YAP exerts its function in cooperation with the TEAD transcription factor. Our data also show that cMYC is a critical factor that acts downstream of the YAP/TEAD complex. Furthermore, we also found that aberrant activation of YAP is sufficient to drive tumorigenic transformation of non-immortalized mouse embryonic fibroblasts. Together our data indicate that YAP can be categorized as a new type of proto-oncogene distinct from typical oncogenes, such as *H-RAS*, whose expression in non-immortalized cells is tightly linked to senescence.

## Introduction

Tumorigenic transformation of normal cells is defined by three criteria: liberation from cell-to-cell contact-mediated growth inhibition, the ability for constant growth under low nutrient conditions, and anchorage-independent growth^1–3^. In normal cells, cell-to-cell contact induces cell cycle arrest, and previous studies demonstrated that inactivation of the YAP transcription factor is central in this process^4,5^. YAP was originally cloned as a Yes-associated protein from chicken^6^. Although the biological and biochemical properties of YAP were initially unknown, genetic analyses with *Drosophila* revealed that YAP (the *Drosophila* homologue is Yorkie) is an important transcriptional regulator of cell growth that is controlled by the Hippo pathway^7^. The Hippo pathway is an evolutionarily conserved signal cascade that is involved in restricting the proliferation of normal cells^8^. In response to cell-cell interaction, e.g., mediated by hemophilic binding of E-cadherin, YAP is phosphorylated by LATS1/2, which are critical kinases in the Hippo pathway. Phosphorylated YAP is retained in the cytosol due to its interaction with 14-3-3 and thus cannot localize in the nucleus. Phosphorylated YAP is recognized by β-TrCP and eventually degraded by SCF ubiquitin ligase-mediated proteolysis^9^. Cell quiescence induced by low serum conditions is achieved by a similar mechanism, in which LATS1/2 is activated by a G-protein-coupled receptor and phosphorylates YAP to target it for ubiquitin-dependent proteolysis^10^.

The YAP transcriptional activator interacts with numerous transcription factors including TEAD1–4^11^, RUNX^12^, KLF4^13^, p73^14^ and TBX5^15^. However, the molecular mechanisms underlying YAP-dependent cell proliferation and cellular transformation remain elusive. Recent chromatin immunoprecipitation-sequence analyses in a human breast cancer cell line demonstrated that the YAP/TEAD complex binds to regions close to the AP-1 binding site in the promoters of genes important for cell cycle progression^16^. These data suggested that YAP associates with TEAD transcription factors and cooperates with the c-Jun/c-Fos complex, at least in part, to exert cellular transformation-inducing activity. Another study demonstrated that the TBX5/YAP complex is involved in colon cancer formation in cooperation with the Wnt signalling cascade^15^. *Drosophila* studies provided further insights into the function of YAP, demonstrating a significant cross-talk between the Hippo signalling cascade and AKT kinase activity^17^. This link was also biochemically demonstrated using the human mammary epithelial fibrocystic disease cell line MCF10A^18^.

The AKT kinase plays a critical role in regulating cell proliferation (reviewed by Manning and Toker 2017^19^ and references therein). Phosphorylation of AKT induced by PI3K activates AKT, and the activated AKT then phosphorylates and inactivates numerous negative regulators of cell proliferation such as FOXO, GSK-3β, and TSC2, leading to cell cycle progression. AKT also promotes cell proliferation by inducing biosynthetic processes through activation of the mTORC1 complex.

Here, we explored the molecular mechanisms by which YAP exerts its transformation-inducing activity and the potential involvement of AKT in this process. Our data show that aberrant activation of YAP is sufficient for inducing tumorigenic transformation of NIH3T3 cells. However, overexpression of YAP did not lead to AKT activation as previously observed with MCF10A cells. Impairment of either YAP or AKT activities did not significantly impact the cell proliferation rate, presumably because of a compensatory role between these two factors when cells are almost free from cell-to-cell contact with neighbouring cells. However, when cells are under dense conditions with abundant cell-cell interactions, AKT is inactivated and cell growth is solely dependent on YAP activity. Our data further revealed that the TEAD transcription factor is crucially involved in this cellular activity of YAP. We also conducted these analyses with mouse embryonic fibroblasts (MEFs), which are non-immortalized normal cells, and found that, similar to NIH3T3 cells, aberrant YAP activation alone is sufficient for inducing tumorigenic transformation in these non-immortalized cells.

## Results

### Overexpression of wild-type YAP or mutant YAP refractory to regulation by the Hippo pathway causes tumorigenic transformation of NIH3T3 cells

Development of cancer is a multistep process, and immortalization is a critical step for tumorigenic transformation. To analyse the role of YAP in tumorigenic transformation, we used NIH3T3 cells. Although NIH3T3 cells are categorized as normal cells, these cells spontaneously transformed into immortalized cells, and acquisition of this phenotype is ascribed to the loss of *Ink4A* and *Ink4B* genes^20^. One advantage in using the NIH3T3 cell line is that we could focus on tumorigenic transformation without the need of factor for immortalization. YAP nuclear localization is required for its ability to promote cell proliferation, and Hippo pathway activation inhibits YAP activity by retaining its localization in the cytosol. In normal cells, including immortalized cells such as NIH3T3 cells, cell-to-cell contact and/or low nutrition conditions are accompanied with activation of the Hippo pathway, leading to cell cycle arrest. We hypothesized that forced expression of YAP in NIH3T3 cells, resulting in dominance of YAP activity over the Hippo pathway, may lead to tumorigenic transformation of NIH3T3 cells.

**Figure 1:**
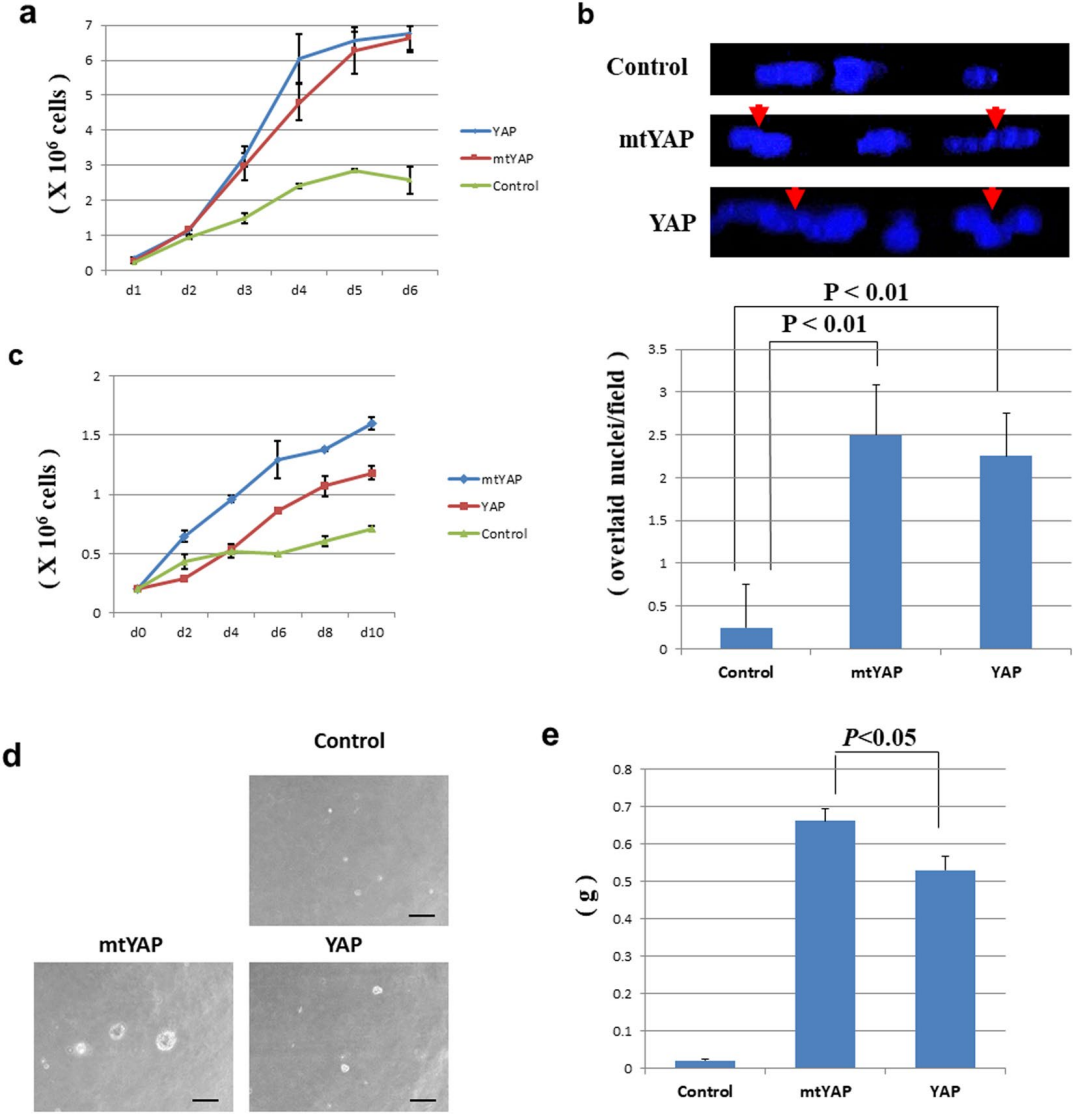
Transformation of NIH3T3 cells by overexpression of wild-type YAP (YAP) or the mutant form (mtYAP). (**a)** Growth curve of NIH3T3 cells stably overexpressing YAP or mtYAP and controls. One hundred thousand cells were plated at day 0 in 10 cm tissue culture dishes. (**b)** Increased overlay of neighbouring cells upon overexpression of YAP. Upper panels: DAPI staining of the indicated cultured cells. Red arrowheads indicate incidences of overlay. Lower panel: quantification of incidence of cell overlay. Overlay events were counted in a region (100 nm on a slide) of cells under a fluorescent microscope. Data were obtained from three independent experiments in which 16 portions were inspected randomly with each sample. (**c)** Cell growth of control NIH3T3 cells and cells stably overexpressing YAP or mtYAP under low nutrient conditions. Growth curves of the indicated cells cultured under low nutrient condition (1% serum). (**d)** Anchorage-independent growth of control NIH3T3 cells and cells stably overexpressing YAP or mtYAP in agarose. The scale bar indicates 100 μm. (**e)** Tumour formation assay. Control NIH3T3 cells or cells stably overexpressing YAP or mtYAP were subcutaneously injected in immune-compromised mice (n = 3). After 4 weeks, tumours were recovered and weighed. Data represent mean ± SD from three independent experiments. Levels of significance were examined by one-way ANOVA.

To address this hypothesis, we stably introduced expression vectors for wild-type YAP in NIH3T3 cells. We also generated stable cell lines expressing the stabilized mutant form of YAP (mtYAP), in which serine at amino acid 112, the residue phosphorylated by the Hippo pathway kinase LATS1/2^21^, was substituted with alanine. Consistent with our hypothesis, we found that NIH3T3 cells overexpressing YAP or mtYAP showed continuous growth even after cells reached confluence (Fig. 1a). The continuous growth of NIH3T3 cells overexpressing either YAP or mtYAP was further supported by the higher frequency of overlay among neighbouring cells (Fig. 1b) and higher number of phospho-histone H3 (ser10)-positive cells compared with control NIH3T3 cells (Supplementary Fig. 1).

Next, we examined the effect of forced expression of YAP and mtYAP on NIH3T3 cells cultured under low nutrition conditions. We found that both YAP and mtYAP enabled NIH3T3 cells to grow continuously even under low nutrition conditions (Fig. 1c). Notably, unlike cells cultured in normal culture conditions, cells overexpressing mtYAP showed a more marked increase in growth under low nutrient conditions compared with cells expressing wild-type YAP (Fig. 1c), suggesting that larger amounts of functional YAP protein, i.e., nuclear localized YAP, are required for counteracting the low nutrition-directed cell cycle arrest compared with the counteraction of cell cycle arrest directed by cell-cell interaction under normal nutrition conditions. To eliminate the possibility that the reduced effects of wild-type YAP under low serum conditions compared with normal culture conditions (10% serum) reflected differences in YAP protein levels between these two culture conditions, we conducted western blot analyses and confirmed that the protein levels of wild-type YAP and mtYAP were not influenced by serum concentration in culture medium (Supplemental Fig. 2).

We next examined whether NIH3T3 cells overexpressing YAP or mtYAP showed anchorage-independent growth. Indeed, both cell lines were able to grow in agarose (Fig. 1d). However, we again noticed that NIH3T3 cells overexpressing wild-type YAP grew much more slowly than cells overexpressing mtYAP (Fig. 1d), indicating that a relatively larger amount of functional YAP protein residing in the nucleus is required for anchorage-independent growth in NIH3T3 cells compared with continuous growth under confluent conditions. Together our data demonstrated that NIH3T3 cells expressing YAP as well as cells expressing mtYAP bear all the characteristics to be defined as transformed cells, i.e., continuous growth in confluent conditions, continuous growth under low nutrition conditions, and anchorage-independent growth.

Since epithelial-mesenchymal transition (EMT) is one of the hallmarks of malignant tumours, we decided to explore whether EMT was induced in YAP-overexpressed NIH3T3 cells. E-cadherin and N-cadherin are representative markers for epithelial and mesenchymal cells, respectively, and are downregulated and upregulated in EMT. Western blot analyses revealed that both overexpressed wild-type YAP and mtYAP resulted in increased and decreased protein levels of E- and N-cadherins, respectively, indicating that YAP overexpression induced EMT (Supplemental Fig. 3).

We next conducted *in vivo* analyses in mice to establish the transformation ability of YAP-overexpressing cells. We subcutaneously injected control NIH3T3 cells or those expressing either wild-type YAP or mtYAP in immunocompromised BALBcA/nu/nu mice and monitored tumor growth. While control NIH3T3 cells did not form appreciable sized tumours in mice, mice injected with cells overexpressing YAP or mtYAP formed tumours (Fig. 1e). Consistent with our *in vitro* results, we found that the tumor formation ability of wild-type YAP-expressing cells was apparently lower compared with mtYAP-expressing cells.

Our above data demonstrated that YAP and mtYAP can induce tumorigenic transformation to NIH3T3 cells. We next inquired whether the observed biological activities of YAP and mtYAP represent effects of the dominance of YAP over the Hippo signalling pathway or if these effects are the result of YAP activity unrelated to the Hippo pathway. To address this question, we examined the Neurofibromatosis 2 (*Nf2*) tumor suppressor gene. MERLIN, the protein encoded by *Nf2*, is an upstream factor that positively regulates the Hippo pathway^22^. MERLIN plays crucial roles in the activation of cell-to-cell contact-mediated Hippo pathway, and this activation is coupled with YAP phosphorylation, leading to its degradation. We knocked down *Nf2* gene expression in normal NIH3T3 cells and found that decreased MERLIN protein levels was accompanied by decreased phosphorylation levels of YAP, leading to stabilization of YAP protein (Supplemental Fig. 4a). We also found that, like NIH3T3 cells overexpressing YAP or mtYAP, NIH3T3 cells deficient for *Nf2* gene expression showed liberation from cell-to-cell contact inhibition and anchorage-independent growth (Supplemental Fig. 4b,c), supporting the notion that the observed effects of overexpressed YAP or mtYAP represent *bona fide* YAP activity intimately linked to the Hippo pathway.

### Overexpression of wild-type YAP or mtYAP in NIH3T3 cells does not affect miR-29a, miR-29b or miR-29c levels

**Figure 2:**
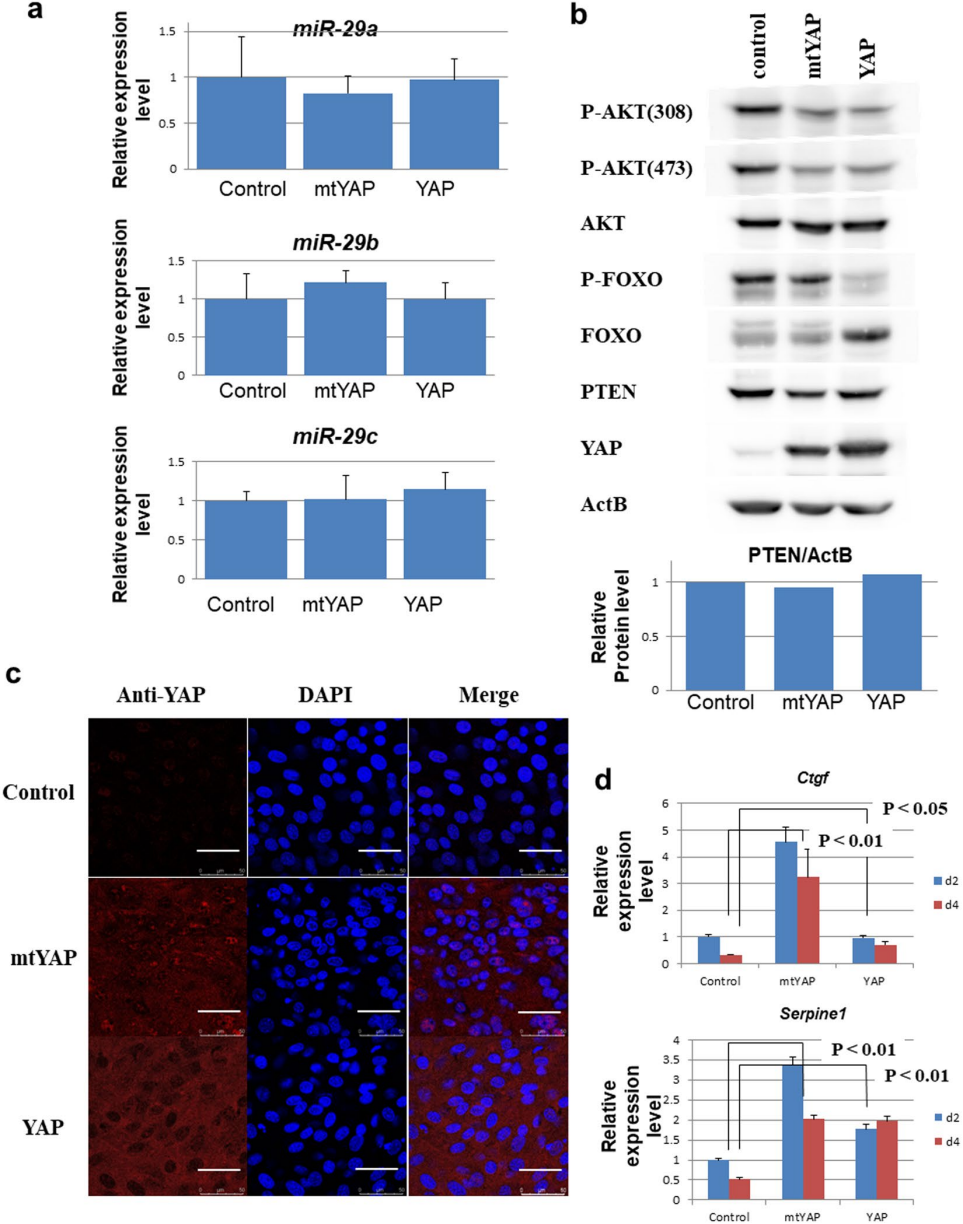
Implication of contribution of TEAD, but not AKT, to YAP activity. (**a)** Expression levels of *miR-29* genes in control NIH3T3 cells and cells expressing YAP or mtYAP under confluent conditions. Expression levels of *miR-29a, miR-29b* and *miR-29c* genes were normalized to those of U6 snoRNA. The expression level of each miRNA in control NIH3T3 cells was arbitrarily set to one. Levels of significance were examined by one-way ANOVA. (**b)** Western blot analyses of the indicated proteins in control NIH3T3 cells and cells expressing YAP or mtYAP under confluent conditions. The graph demonstrates the relative PTEN protein levels normalized using ActB protein levels as an internal control. (**c)** Immunocytochemical analyses of YAP in control NIH3T3 cells and cells expressing YAP or mtYAP under confluent conditions. (**d)** Expression levels of *Ctgf* and *Serpine 1* in control NIH3T3 cells and cells expressing YAP or mtYAP under subconfluent (2 days after plating, d2) or confluent conditions (4 days after plating, d4). The expression level of each gene in control NIH3T3 cells was arbitrarily set to one. Data represent mean ± SD from three independent experiments. Levels of significance were examined by one-way ANOVA.

Tumaneng *et al*.^18^ recently reported that AKT activation is crucially involved in the transformation inducing activity of YAP in MCF10A cells. YAP interacts with the TEAD transcription factor, and the YAP-TEAD complex binds the promoter of the *miR-29a*, *miR29b* and *miR-29c* genes to promote their expression. Elevation of these miRNAs leads to the activation of AKT by inhibiting the translation of mRNA for PTEN that is tightly linked to inactivation of AKT through PTEN-mediated dephosphorylation of phosphatidylinositol (3,4,5)-triphosphate. Therefore, we examined whether NIH3T3 cells overexpressing YAP or mtYAP showed increased levels of the *miR-29* genes. However, we did not detect any differences in levels of the *miR-29* genes in NIH3T3 cells overexpressing either YAP or mtYAP compared with control cells under confluent conditions (Fig. 2a). Phosphorylation levels of AKT, which indicates AKT activation, were not elevated in cells with overexpression of YAP or mtYAP, and instead AKT phosphorylation was decreased in cells cultured under confluent conditions (Fig. 2b). We also did not observe an elevation in phosphorylated FOXO3, one of the major targets of AKT, or decline in PTEN protein levels in NIH3T3 cells overexpressing YAP or mtYAP compared with controls.

To eliminate the possibility that the absence of elevation in *miR-29* expression was due to the failure of introduced YAP and mtYAP to exert expected functions, we examined the subcellular localization of the exogenous YAP proteins by immunocytochemistry. While wild-type YAP localized mainly in the cytosol, with some nuclear localization, we found marked nuclear localization of mtYAP in NIH3T3 cells even under confluent conditions (Fig. 2c).

We next examined the expression levels of major YAP/TEAD complex target genes in NIH3T3 cell lines stably expressing wild-type YAP or mtYAP. Previous studies showed that mtYAP significantly induced the expression levels of major YAP/TEAD complex target genes, *Ctgf* and *Serpine 1* genes^23^. Our data demonstrated similar activities of mtYAP in inducing *Ctgf* and *Serpine 1* (Fig. 2d). In contrast, wild-type YAP had little effect on *Ctgf* levels but induced *Serpine 1*, although slightly less efficiently than mtYAP. However, overexpressed wild-type YAP appeared to counteract confluent condition-dependent downregulation of expression of both YAP/TEAD target genes, in contrast with data from normal NIH3T3 cells in which the expression levels of these genes were significantly reduced when cells shifted from subconfluent to confluent growth. Together, our data indicate that the YAP/TEAD complex induces *Ctgf* and *Serpine 1* gene expressions, but not *miR-29* gene levels, in NIH3T3 cells.

### YAP promotes cell cycle progression independent of AKT activity

**Figure 3:**
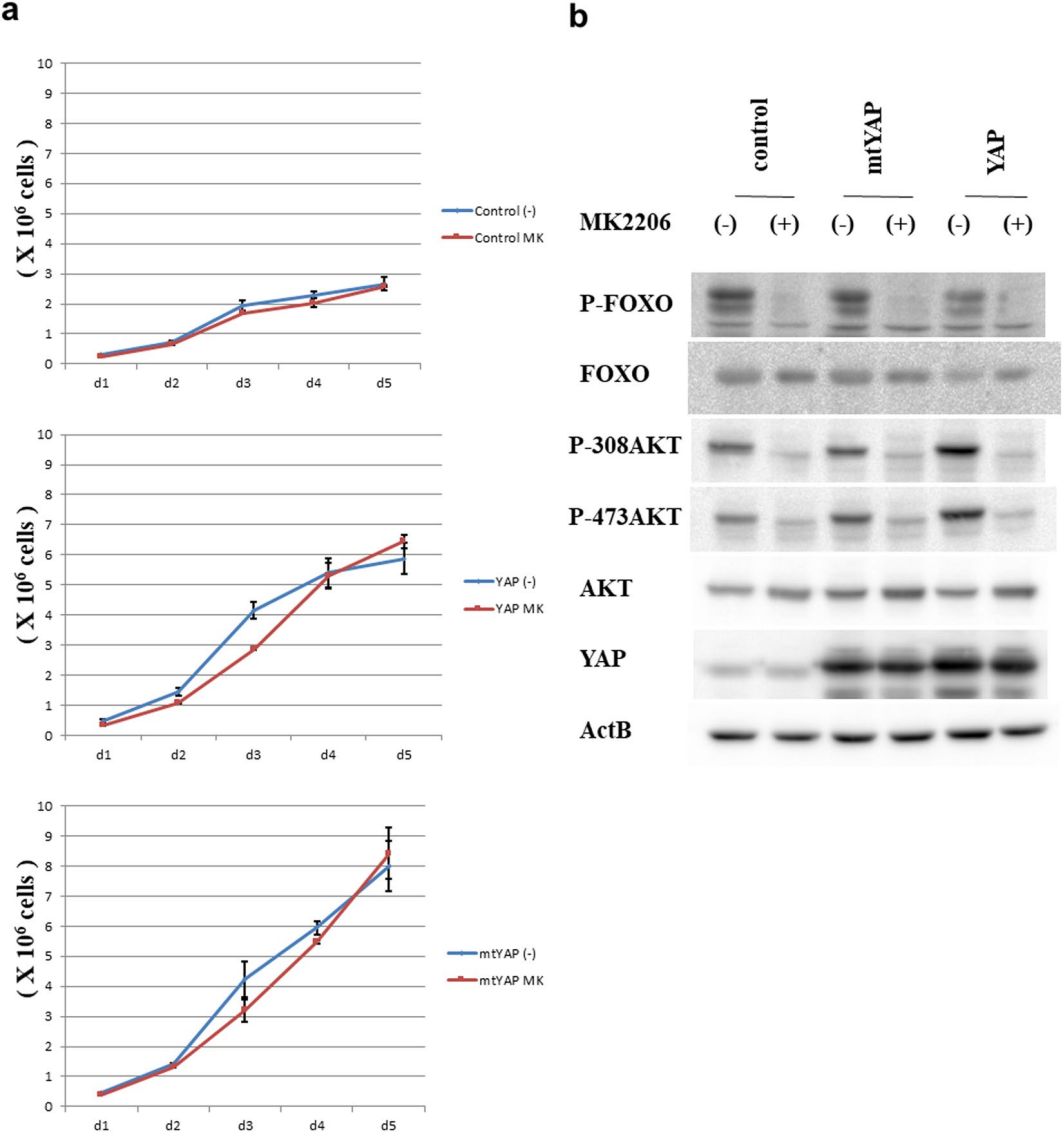
Inhibition of AKT activity has no effect on cell growth of NIH3T3 cells expressing YAP or mtYAP and controls. (**a)** Growth assay in control NIH3T3 cells and cells overexpressing YAP or mtYAP cultured with or without AKT inhibitor (MK2206, 1 μM). (**b)** Western blot analyses of the indicated proteins in control NIH3T3 cells and cells expressing YAP or mtYAP cultured with or without AKT inhibitor (MK2206, 1 μM).

Our data so far demonstrated that YAP is sufficient for tumorigenic transformation of NIH3T3 cells, but the *miR29*-PTEN-AKT axis is not involved in this transformation. To further confirm that AKT was not involved in YAP-mediated transformation, we treated cells with an AKT inhibitor, MK2206, and monitored cell growth. We observed no noticeable difference in the growth curves between NIH3T3 cells treated with MK2066 and controls (Fig. 3a). We also did not detect any significant differences in NIH3T3 cells overexpressing YAP or mtYAP with MK2206 treatment compared with controls. To confirm that MK2066 was effectively inhibiting AKT in NIH3T3 cells, we performed western blot analyses (Fig. 3b). Phosphorylation levels of both AKT and FOXO were significantly decreased upon treatment with MK2066 in all three cell lines, confirming that MK2066 showed expected efficacy. AKT also shows kinase-independent activity^24^, and thus all AKT activities are not inhibited by the treatment of MK2066. Therefore, we addressed the possibility that AKT may exert kinase-independent effects on YAP activity. We used an allosteric inhibitor, SC66, which facilitates ubiquitination of AKT and its subsequent degradation^25^. Western blot analyses confirmed that SC66 treatment led to the reduction of AKT protein level in a dose-dependent manner that was accompanied by decreased phosphorylation levels of FOXO3 (Supplemental Fig. 5a). We thus treated control NIH3T3 cells and cells overexpressing mtYAP or YAP with SC66 and monitored cell growth. Like MK2066, SC66 showed no noticeable effect on cell proliferation (Supplemental Fig. 5b), indicating that overexpressed YAP and mtYAP can elevate the cell proliferation rate in NIH3T3 cells independent of AKT. Since AKT is a crucial regulator of cell proliferation, we speculate that YAP and AKT function in parallel as dual independent driving forces for cell proliferation, and therefore inactivation of either YAP or AKT may be almost completely compensated by the other for sustaining the growth rate of normal cells.

We next examined whether a synergistic effect of AKT and YAP on cell cycle progression would become evident if YAP and AKT are both aberrantly activated. We introduced myristoylated-AKT (Myr-AKT), which functions as a constitutively active kinase without dependency on PI3K^26^, into control and mtYAP-overexpressing NIH3T3 cells. Western blot analyses showed that Myr-AKT was efficiently activated, i.e., phosphorylated at both Thr308 and Ser473, in cell lines (Supplemental Fig. 7a). Examination of co-expression of Myr-AKT and mtYAP on cell cycle progression was hampered due to the unexpected negative effect of forced expression of Myr-AKT on cell proliferation under regular culture conditions (Supplemental Fig. 7b). However, we observed a synergistic effect of co-expression of both Myr-AKT and mtYAP on cell growth in agarose compared with the effect of either Myr-AKT or mtYAP alone (Supplemental Fig. 7c). These results suggest that Myr-AKT and mtYAP function cooperatively at least in the context of anchorage-independent growth.

### *cMyc* is a direct target of YAP

**Figure 4:**
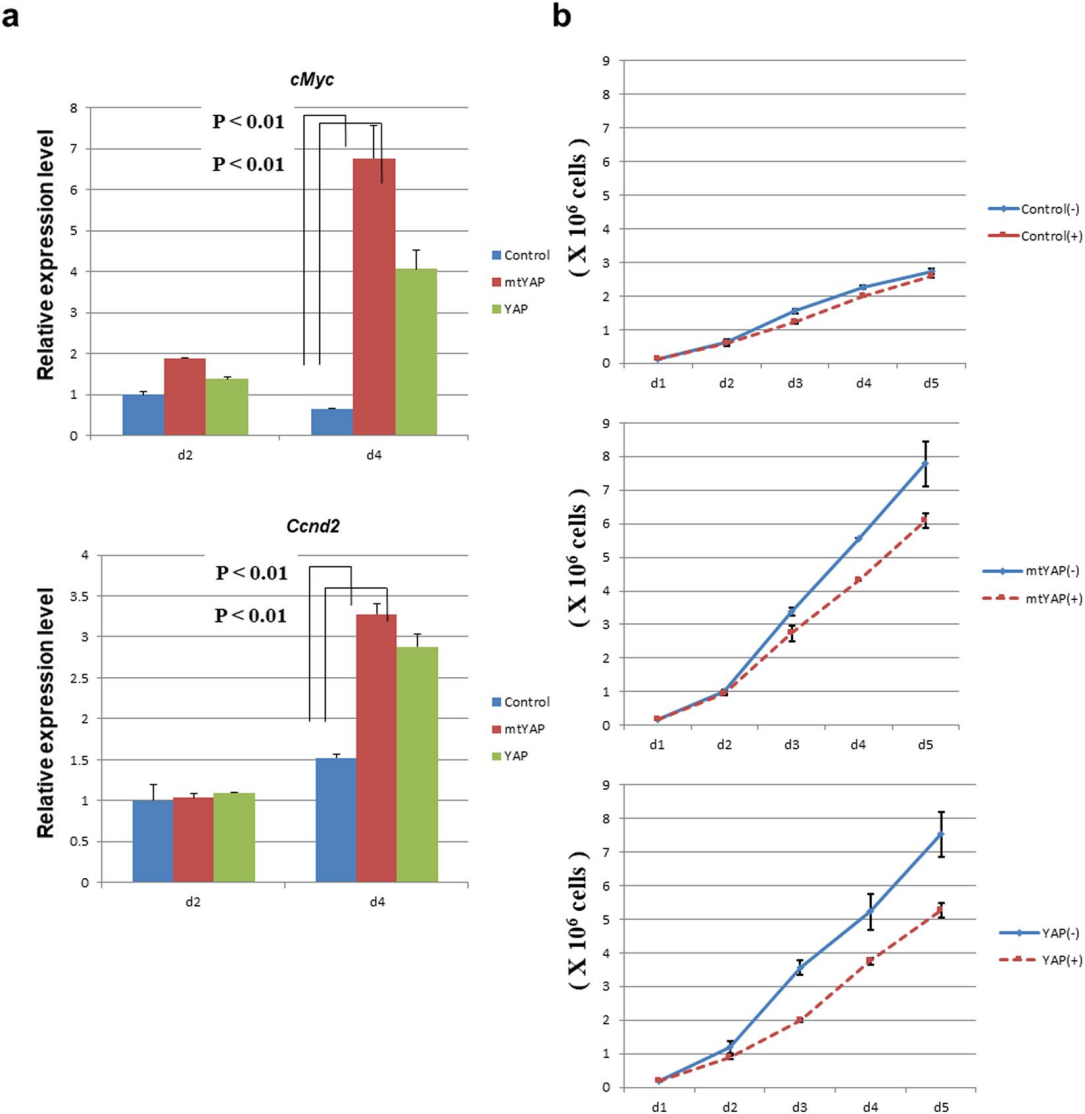
Involvement of cMYC in the growth of NIH3T3 cells expressing YAP or mtYAP under confluent conditions. (**a)** Expression levels of *cMyc* and its major target *Ccnd2* in control NIH3T3 cells and cells expressing YAP or mtYAP. RNAs were prepared from the cells at day 2 or 4 (d2 and d4, respectively) and expression levels of *cMyc* and *Ccnd2* were examined by quantitative PCR. The expression level of each gene in control NIH3T3 cells was arbitrarily set to one. Levels of significance were examined by one-way ANOVA. (**b)** Growth curve of control NIH3T3 cells and cells expressing YAP or mtYAP cultured with or without cMYC inhibitor (10058-F4, 64 μM).

Although a link between aberrant activation of YAP and acceleration of cell division has been well documented, the exact YAP target genes involved in YAP-mediated regulation of cell cycle progression are unknown. Chromatin immunoprecipitation sequence analyses in MDA-MB-231 breast cancer cells indicated *cMyc* as one of the major effectors for YAP^16^. Therefore, we examined whether cMYC functions as one of the major downstream effectors of YAP also in NIH3T3 cells. Expression levels of both *cMyc* and *Ccnd2*, well-known targets of cMYC, were not appreciably altered due to overexpression of YAP or mtYAP at day 2, in which cells are largely in subconfluent conditions (Fig. 4a). However, at day 4 when cells are largely in confluent conditions, significantly higher expression levels of *cMyc* and *Ccnd2* were evident in NIH3T3 cells overexpressing YAP or mtYAP compared with control NIH3T3 cells (Fig. 4a). These results indicate that *cMyc* is a target of YAP when cells are under confluent conditions. Consistent with this notion, treatment of NIH3T3 cells overexpressing YAP or mtYAP with the 10058-F4 cMYC inhibitor, which impairs the interaction of cMYC with its partner protein MAX^27,28^, caused reduced cell proliferation in cells under confluent conditions but not in subconfluent conditions (Fig. 4b). In contrast, 10058-F4 did not affect cell proliferation of control NIH3T3 cells irrespective of cell density. At present, we do not know why overexpressed YAP and mtYAP were not involved in transcriptional activation of *cMyc* when cells were under subconfluent conditions. Since cMYC is the most powerful driving force for cell proliferation, we speculate that cells under subconfluent condition bear certain molecular mechanisms that prevent overexpressed YAP to exert its maximum activity to avoid hyper-activation of cMYC in conditions in which endogenous YAP and other molecules that promote cell proliferation are already intensively operating.

### TEAD is critical in supporting YAP-dependent cell cycle progression

Numerous factors such as Klf4, RUNX, TBX3 and p73 are binding partners for YAP. However, the interacting transcription factor that is critical for supporting the cell cycle progression activity of YAP has not been determined. Recent chromatin immunoprecipitation sequence analyses in MDA-MB-231 breast cancer cells showed elevated expression of multiple genes involved in cell cycle progression from simultaneous bindings of YAP and TEAD4 in their gene promoters^16^. Therefore, we next examined whether the YAP/TEAD complex is critical for cell cycle progression in NIH3T3 cells. We constructed an expression vector for the dominant-negative TEAD protein (DN-TEAD). The TEAD1 Y408H mutation was first identified as a causative mutation of Sveinsson chorioretinal atrophy^29^, and this mutation impairs the interaction between YAP and TEAD^30^. To render the function of DN-TEAD switchable, we produced an expression vector for DN-TEAD fused to the hormone-binding domain of oestrogen receptor (DN-TEAD-ERT). We generated stable transformants expressing DN-TEAD-ERT in NIH3T3 cells as well as in YAP- and mtYAP-expressing NIH3T3 cells. Western blot analyses confirmed that protein levels of DN-TEAD-ERT in control NIH3T3 cells and those overexpressing YAP or mtYAP were comparable (Supplemental Fig. 6a). We treated these cells with 4-hydroxytamoxifen (0.5 μM) for 4 days, which was effective to stabilize and accumulate the fusion protein in the nucleus (Supplementary Fig. 6b).

**Figure 5:**
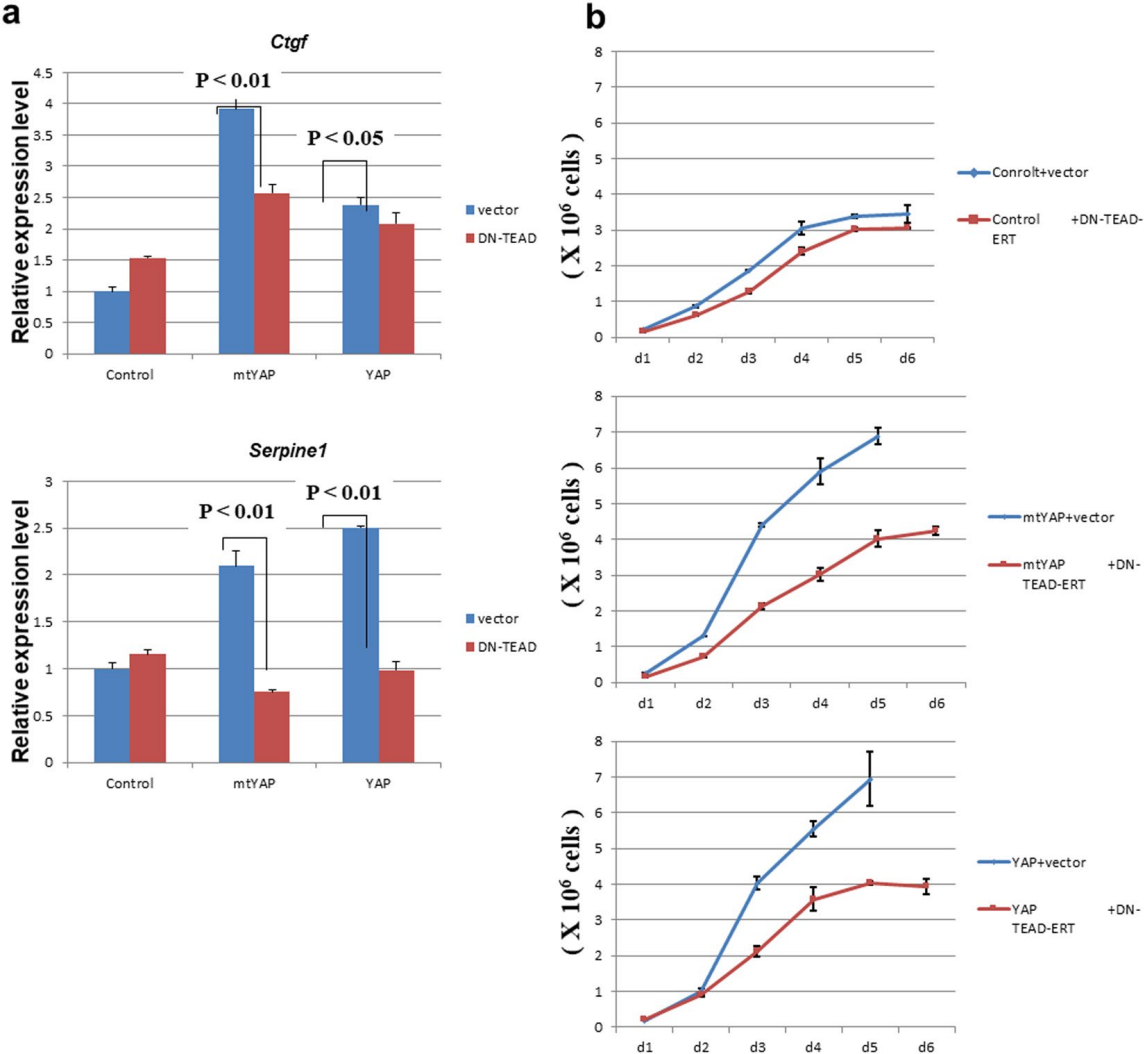
Involvement of TEAD transcription factor in the YAP-mediated transformation of NIH3T3 cells. (**a)** Effect of DN-TEAD-ER fusion protein on expression levels of YAP/TEAD target genes. Quantitative PCR of YAP/TEAD target genes *Ctgf* and *Serpine 1* in control NIH3T3 cells and cells overexpressing YAP or mtYAP in which an expression vector for DN-TEAD-ER fusion protein or empty vector had been stably introduced. The expression level of each gene in control NIH3T3 cells was arbitrarily set to one. Student’s t test was used to determine levels of significance. **b** Growth curve analyses of the indicated cells.

We first measured the expression levels of *Ctgf* and *Serpine1* in cells treated with 4-hydroxytamoxifen. Expression of DN-TEAD-ERT caused decreased levels of *Ctgf* and in particular *Serpine1* to levels close to controls (Fig. 5a). DN-TEAD-ERT did not cause noticeable effects on cell growth in control NIH3T3 cells in both subconfluent and confluent conditions, suggesting that AKT, which should be active even in DN-TEAD-ERT-expressing cells, compensates for the impaired function of the YAP/TEAD complex (Fig. 5b). In NIH3T3 cells overexpressing YAP or mtYAP cultured in subconfluent conditions, DN-TEAD-ERT did not affect cell growth. However, in confluent conditions, DN-TEAD-ERT caused reduced growth (Fig. 5b), indicating that TEAD plays a pivotal role in supporting the function of YAP for sustaining the continuous growth of cells under confluent conditions.

### Exploration of the molecular functions of YAP and mtYAP through global gene expression analyses

**Figure 6:**
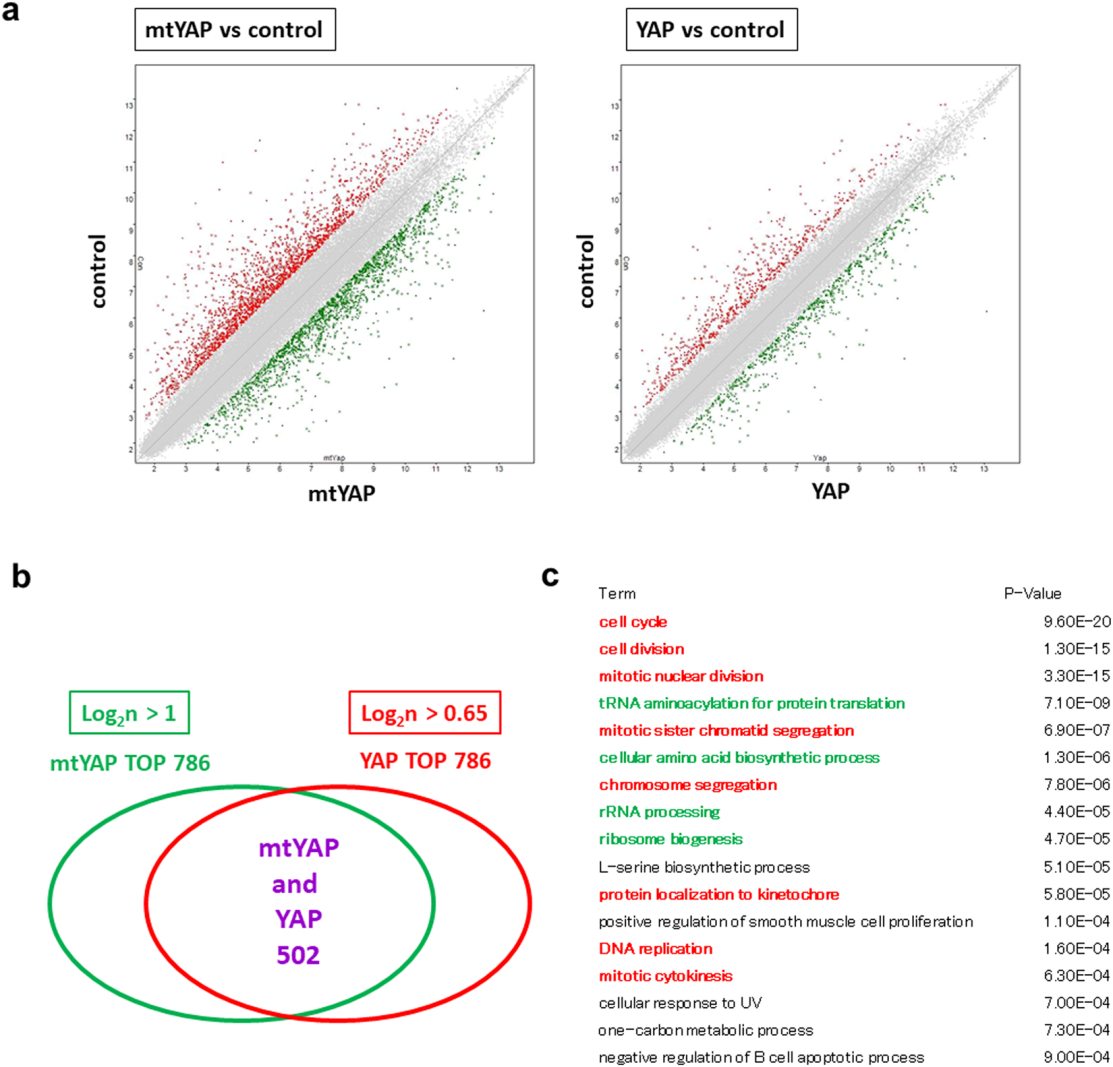
Mutant YAP is more potent than wild-type YAP in altering gene expression in NIH3T3 cells. (**a)** Scattered plots of global expression data in control NIH3T3 cells and cells expressing YAP or mtYAP. Global expression data from control NIH3T3 cells were compared with those expressing mtYAP (left panel) or wild-type YAP (right panel). Green and red dots indicate genes up- and down-regulated, respectively, more than two-fold due to expression of YAP or mtYAP. (**b)** Venn diagram showing the relationship among the top 786 genes whose expression levels were elevated by 1.65- and 2-fold from forced expression of YAP and mtYAP, respectively. (**c)** Significantly enriched GO terms of 502 genes that were commonly activated by both YAP and mtYAP. Terms directly linked to cell growth are indicated with in red font, while terms that are relatively related to cell growth are indicated with in green font.

To explore the molecular basis underlying the promotion of cell growth by YAP and mtYAP in NIH3T3 cells even under confluent conditions, we conducted DNA microarray analyses. Our analyses revealed that 786 genes showed more than two-fold higher expression levels in cells overexpressing mtYAP compared with control cells (Fig. 6a). Relatively fewer genes (280 genes) showed increased expression levels by more than two-fold in cells expressing wild-type YAP compared with controls. Therefore, we lowered the criterion of cut-off (more than 1.65-fold instead of 2-fold) to select the same number of genes (786 genes) as a gene set from this group. We then examined the overlapping genes between the two gene sets. Among the 786 genes, 502 genes were common in both gene sets (Fig. 6b, Supplemental Table 4). Notably, *cMyc* was one of the 502 genes. The genes were assigned to gene ontology (GO) classification to assess the overall molecular functions of these genes. GO terms related to the cell cycle were significantly enriched (Fig. 6c), indicating that the genes generally known to function for cell cycle progression as driving forces for cell growth of NIH3T3 cells become able to function persistently even after the cells are subjected to confluent conditions by overexpression of wild-type YAP or mtYAP.

### Tumorigenic transformation of non-immortalized MEFs by aberrantly activated YAP alone

Because NIH3T3 cells are spontaneously transformed (immortalized) cells, we next conducted experiments using mouse embryonic fibroblasts (MEFs) as non-immortalized primary cultured cells that show senescence after relatively long culture^31^. Introduction of an oncogene alone, e.g., activated *H-RasV12G*, is not sufficient for inducing tumorigenic transformation in MEFs and other non-immortalized cells and requires an additional factor, such as SV40 large T-antigen (LT), that confers immortalization. Forced expression of an oncogene alone does not induce tumorigenic transformation of MEFs, but instead rapidly induces senescence in these cells^32^. Thus, we generated MEFs stably expressing LT together with mtYAP as well as MEFs expressing either LT or mtYAP alone. Since the senescence of normal cells is induced by p53 stabilization^33^, we examined p53 levels. Control MEFs showed accumulated p53 at passage 32, while no expression was detected at passage 7 (Supplementary Fig. 8), confirming that these are non-immortalized cells that can undergo senescence by prolonged culture.

**Figure 7:**
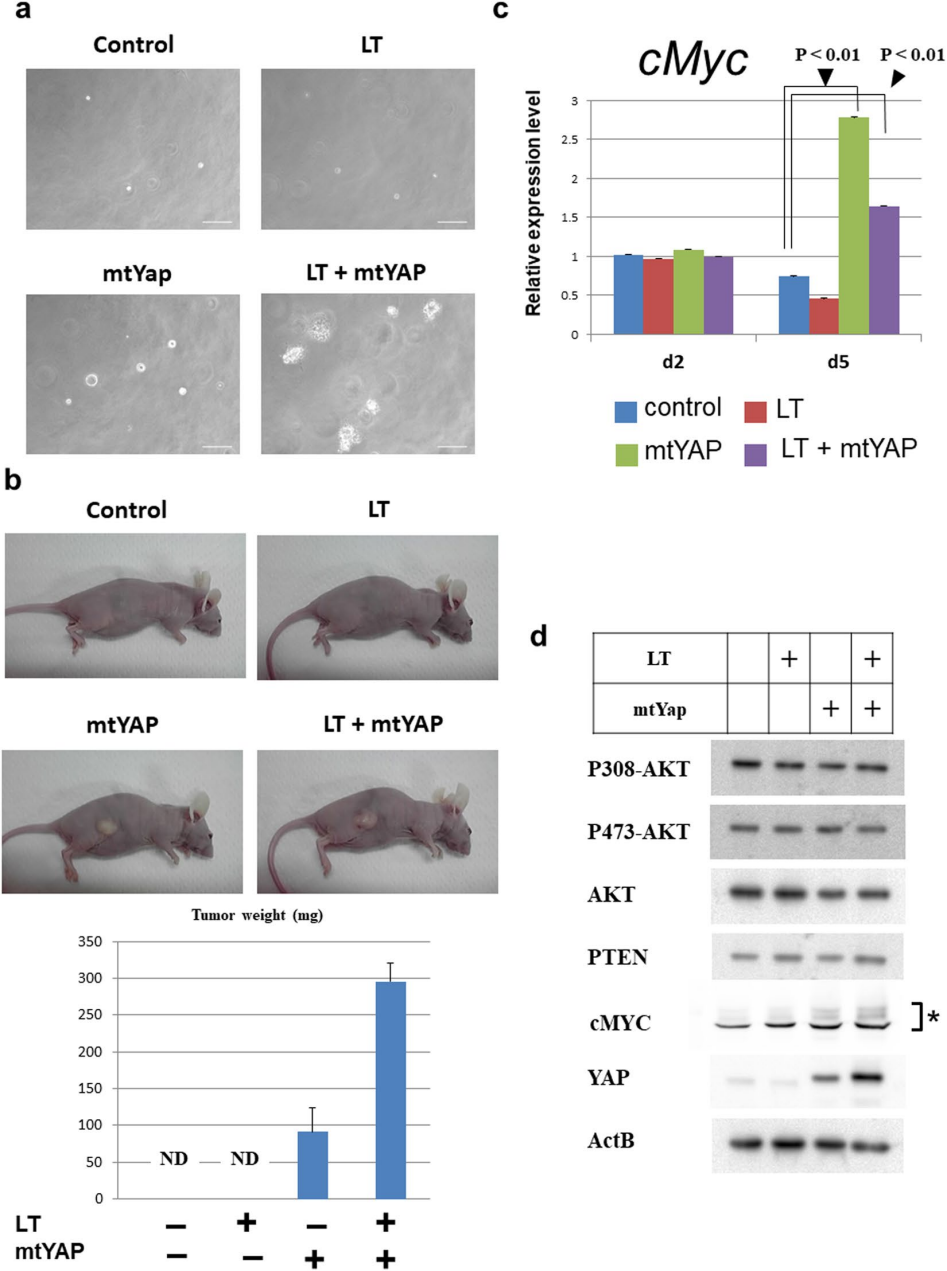
Tumorigenic ability of MEFs by aberrant YAP activation alone. (**a)** Anchorage-independent growth of control MEFs and cells expressing LT and/or mtYAP. (**b)** Tumor formation ability of control MEFs and cells expressing LT and/or mtYAP. (**c)** Examination of *cMyc* expression levels in control MEFs and cells expressing LT and/or mtYAP under sparse (d2) and confluent conditions (d5). The expression level of *cMyc* gene in control NIH3T3 cells was arbitrarily set to one. Levels of significance were examined by one-way ANOVA. (**d)** Western blot analysis in control MEFs and cells expressing LT and/or mtYAP. cMYC protein was detected as multiple bands that may reflect posttranslational modifications.

We next examined the tumorigenic property of MEF-based cell lines and first examined anchorage-independent growth ability. Control MEFs and those expressing LT alone did not form colonies in agarose, whereas MEFs co-expressing mtYAP and LT grew and formed colonies in agarose (Fig. 7a). These results were consistent with our data from NIH3T3 cells, as MEFs expressing LT may functionally correspond to the spontaneously immortalized NIH3T3 cells. However, MEFs expressing mtYAP alone grew in agarose and formed colonies, although the colony-forming speed was much lower than in cells expressing both mtYAP and LT.

We next examined the tumorigenic ability of MEF-based cell lines by subcutaneously transplanting these cells into immunocompromised BALBcA/nu/nu mice. Control MEFs and LT-expressing MEFs did not generate tumours (Fig. 7b). Consistent with our data on anchorage-independent growth, MEFs expressing mtYAP alone as well as MEFs expressing mtYAP and LT formed tumours, although the tumor growth rate from MEFs expressing mtYAP was lower than that from MEFs expressing mtYAP and LT (Fig. 7b). Furthermore, no significant difference was observed in *cMyc* expression level among the four cell lines under sparse conditions (day 2). However, significantly higher expression of *cMyc* was evident in MEFs overexpressing mtYAP alone and those overexpressing both mtYAP and LT under confluent conditions (day 4), indicating that cMYC functions as one of the important downstream effectors of mtYAP in MEFs (Fig. 7c). We also found higher levels of cMYC protein in MEFs with exogenous mtYAP compared with cells without mtYAP (Fig. 7d). These results again support the notion that aberrantly activated YAP exerts tumorigenic transformation-promoting activity through transcriptional activation of cMYC with TEAD transcription factor. Finally, we examined the phosphorylated status of AKT in these cell lines under confluent conditions. Consistent with the results in NIH3T3 cells, no AKT activation was observed due to forced expression of mtYAP and/or LT (Fig. 7d).

## Discussion

AKT functions as a positive regulator of cell proliferation and promotes cell growth through its phosphorylation of specific downstream targets^34–38^. In normal cells, the kinase activities of AKT, which are in parallel to its phosphorylation levels, are strictly controlled by the RAS-PI3K cascade and PTEN phosphatase, which act as positive and negative regulators for AKT, respectively^19^. Consistent with the intrinsic involvement of the RAS-PI3K-AKT axis and Hippo pathway in the regulation of cell growth, mutations in the components of both pathways are frequently observed in many cancer types. In the RAS-AKT cascade, numerous gain-of-function mutations in *RAS* and *AKT* genes and loss-of-function mutations in genes such as *PTEN* have been reported in cancers^39^. Likewise, in the Hippo pathway, a negative regulator of proliferation in normal cells, loss-of-function mutations in *NF2* and *LATS1/2* genes and gene amplification of *YAP* gene as a gain-of-function mutation in cancers have been reported^16^. Recently, a cross-talk between these two pathways was reported. Tumaneng *et al*.^18^ demonstrated that YAP activates transcription of the *miR-29* gene in MCF10A human mammary fibrosis cells, and *miR-29* activates AKT through its inhibition of PTEN. However, our data showed that constitutive activation of YAP causes transformation in NIH3T3 cells without modulating AKT activity, suggesting that the link between YAP and AKT for cell growth is species-specific or cell type-specific.

Aberrant activation of YAP in cancer is caused by two mechanisms: one mechanism involves inactivation of the Hippo pathway, and the other mechanism involves increased YAP protein levels from gene amplification^4^. Although these two mechanisms result in a functional dominance of YAP over the Hippo pathway, the cellular consequences of these mechanisms are distinct. Loss-of-function mutations in the components of the Hippo pathway allow YAP to constitutively reside in the nucleus even under confluent conditions, whereas overexpressed YAP due to gene amplification shows inefficient nuclear localization, especially when cells are under confluent conditions, due to the normally operating Hippo pathway^21^. Our experimental systems in NIH3T3 cells overexpressing YAP or mtYAP, in which the serine target of LATS1/2 was substituted with alanine, recapitulate these two cancer types in which aberrantly activated YAP is crucially involved: YAP overexpression corresponds to *YAP* gene amplification, while expression of mtYAP that is refractory to regulation by the Hippo pathway corresponds to Hippo pathway inactivation. Our data suggest that overexpression of wild-type YAP and mtYAP in NIH3T3 cells represents a *bona fide* model of the two YAP-related cancer types. Indeed, mtYAP showed enhanced nuclear accumulation compared with wild-type YAP. Our results also indicated that due to increased nuclear localization, mtYAP exerted more prominent effects compared with wild-type YAP regarding cell growth promotion. Thus, our data indicate that activation, and stabilization, of YAP through inactivation of the Hippo pathway is more efficient in conferring transformation characteristics in normal cells than only YAP overexpression. Moreover, DNA microarray analyses revealed more pronounced effects of mtYAP on altering gene expression than wild-type YAP. However, the dataset comparisons suggested a similarity in the molecular mechanisms of wild-type YAP and mtYAP in inducing transformation in normal cells. Our data also show that cMYC is one of the major downstream effectors of YAP. Interestingly, a recent study showed that cMYC and YAP cooperatively activate genes that are critical for cellular proliferation^40^. Together these results indicate an interplay between YAP and cMYC in multiple aspects of tumorigenic transformation of immortalized and non-immortalized normal cells.

Previous studies have established that the expression of an oncogene such as activated *H-RAS* alone does not lead to tumorigenic transformation but instead induces senescence in non-immortalized mouse primary cells such as MEFs^32^. These primary cells require co-expression of a gene that induces immortalization together with expression of an oncogene for tumorigenic conversion. Since *YAP* is classified as an oncogene, we speculated that, like H-RAS, aberrantly activated YAP alone may induce tumorigenic transformation of immortalized NIH3T3 cells but not non-immortalized MEFs. However, expression of mtYAP alone was sufficient for inducing tumorigenic transformation in MEFs as well as in NIH3T3 cells (Fig. 7a,b), indicating that YAP not only exhibits oncogenic activity, but also shows immortalization-inducing activity. Alternatively, YAP may somehow mask the requirement of immortalization activity for tumorigenic transformation of non-immortalized primary cells. Although the precise molecular basis underlying the cellular transformation by YAP still remains elusive, we hope that our study provides important findings towards complete understanding of this process.

## Methods

### Antibodies and reagents

Antibodies used for western blotting and immunocytochemical analyses were as follows: anti-AKT (#9272), anti-YAP (#12395), anti-FOXO1 (#2880), anti-PTEN (#9559), anti-p53 (#2524), anti-HA (H3663), anti-β-actin (sc47778), anti-phosphorylated YAP (#13008), anti-phospho histone H3 (Ser10) (#9701), anti-phosphorylated AKT (threonine 308, #2965; serine 473, #9271), anti-phosphorylated FOXO1 (threonine 24 and 32, #9464), anti-cMYC (#5605), anti-E-cadherin (#3195), anti-N-cadherin (#14215) and anti-Merlin (#12888). All antibodies were purchased from Cell Signaling Technology, except for antibodies against HA-tag and β-actin, which were obtained from Sigma-Aldrich and Santa Cruz Biotechnology, respectively. The AKT inhibitor Mk2206 and SC66 from Selleckchem were used at final concentrations of 1 μM and 2 μg/ml respectively. The cMYC inhibitor 10058-F4 from Millipore was used at a final concentration of 64 μM. Phalloidin-iFluor 488 conjugate from Cayman Chemicals was used according to the manufacturer’s protocol.

### Cell culture

NIH3T3 cells were cultured with DMEM supplemented with 10% calf serum and MEFs were cultured with DMEM supplemented with 10% fetal calf serum. Both cell lines were cultured at 37 °C in a 5% CO_2_ incubator. MEFs were prepared from 14.5 dpc embryos of a C57BL/6 J mouse.

### Plasmid construction and establishment of stable cell lines

To construct retrovirus vectors of pMCs-IRES-Puro carrying mouse wild-type YAP, mutant YAP (S112A, mtYAP) or SV40 large T-antigen (LT), the IRES-EGFP region in pMCs-IRES-EGFP (Addgene) was replaced by the IRES-Puro fragment. The Gateway cassette B (ThermoFisher) was inserted in this plasmid. The coding sequence of mouse YAP, mtYAP, or LT followed by Flag-tag sequence was inserted into the pENTR1A vector (ThermoFisher). YAP, mtYAP and LT cDNAs were individually inserted into pMCs-Gateway cassette B using LR clonase (ThermoFisher). pMCs-IRES-Puro carrying either mouse wild-type YAP, mtYAP or LT or empty vector was individually transfected into Plat-E cells^41^ using FuGENE transfection reagent (Promega). At 2 days post-transfection, virus particle-containing medium was recovered and used to infect NIH3T3 cells or MEFs plated in cell culture dishes. At 2 days post-virus infection, virus-infected NIH3T3 cells or MEFs were selected with puromycin at a concentration of 1.5 μg/ml. The surviving cells were cultured and used for subsequent biological and biochemical analyses.

To construct the lentiviral expression vector for dominant negative mouse TEAD1 fused to HA-tagged hormone binding domain of human oestrogen receptor (DN-mTEAD1-HA-ERT2-IH), the IRES-hygromycin resistant gene fragment was first inserted into the CSII vector. The cDNA encoding the dominant negative TEAD1 mutant (Y431H), which is flanked by HA-tag and ERT2 sequences, was inserted upstream of the IRES-hygromycin resistant gene. The resultant vector (CSII-DN-mTEAD1-HA-ERT2-IH) was transfected into 293FT cells using FuGENE transfection reagent together with three packaging plasmids (pPL1, pPL2, and pVSVG). At 2 days post-transfection, virus particle-containing medium was recovered and used to infect NIH3T3 cells. On the following day, infected cells were cultured in the presence of hygromycin (300 μg/ml) for selection. Selected cells were cultured in the presence of hygromycin and used for subsequent analyses.

To construct the shRNA lentivirus vector for *Nf2* knockdown, the three sets of double-strand oligonucleotides shown in Supplemental Table 2 were each inserted into AgeI and EcoRI sites of the pLKO.1-puro vector^42^. The resultant vectors (sh1, sh2, or sh3) were used to generate lentiviruses as above, and the generated viruses were used to establish stable cell lines by infection of NIH3T3 cells followed by selection with puromycin (1.5 μg/ml).

### Anchorage-independent growth analyses

Cells suspended in 0.33% agarose with medium were poured on 0.5% agarose with medium. Cells in agarose were cultured in 5% CO_2_ at 37 °C for 4 weeks and then photos were taken under a microscope.

### Immunocytochemical analyses

Immunocytochemical analyses were conducted as described previously^43^. The primary and secondary antibodies used for immunochemical analyses are listed in Supplementary Table 1.

### Tumor formation assay

BALBcA/nu/nu mice were obtained from CLEA Japan, Inc. Two million cells (NIH3T3 cell lines or MEFs) were subcutaneously transplanted in 6–8-week-old BALBcA/nu/nu mice. This study was conducted in strict accordance with the international and institutional guidelines. The protocol for all animal experiments conducted in this study was approved by institutional review boards on the Ethics of Animal Experiments of the Saitama Medical University (permission number 261).

### Quantitative RT-PCR

cDNAs were generated by reverse transcription of RNA from NIH3T3 control or stable cells according to the manufacturer’s instructions. Quantitative RT-PCR was conducted with SYBR Green-based reactions on the StepOnePlus real-time PCR system (ThermoFisher). Primers were designed by the program of Universal ProbeLibrary Assay Design Center (Roche Lifescience). PCR primers are listed in Supplementary Table 2. Data were normalized to *ß-actin* expression levels.

To evaluate levels of miR-29a, miR-29b and miR-29c, total RNA including miRNA was purified from cells using the miRNeasy mini kit (QIAGEN). Reverse transcription was conducted using the TaqMan small RNA assay kit (ThermoFisher). Levels of miR-29a, miR-29b and miR-29c were quantified by TaqMan-based reactions using the StepOnePlus real-time PCR system and normalized to U6 snoRNA.

### Western blot analyses

Whole cell extracts were prepared in lysis buffer (50 mM Tris-Cl pH 7.5, 150 mM NaCl, 0.1% SDS, 0.5% deoxycholate), protein inhibitor cocktail (Nacalai, Japan) and phosphatase inhibitor cocktail (Pierce). Western blot analyses were conducted as described previously^43^. The primary antibodies used for western blotting are listed in Supplementary Table 1.

### Microarray analysis

Biotin-labelled cRNAs were synthesized and hybridized to Affymetrix GeneChip Mouse Genome 430 2.0 arrays according to the manufacturer’s instructions. Microarray expression data were background subtracted and normalized with the robust multiarray analysis method using statistical software R. Gene ontology (GO) analyses were conducted using DAVID (https://david.ncifcrf.gov).

### Statistical analyses

Statistically significant differences among three groups were examined by one-way ANOVA. The significant level was compensated by means of Bonferroni correction.

## Supplementary information

supplemental Figures and Tables

## Data Availability

DNA microarray data generated in this study were deposited in NCBI’s Gene Expression Omnibus under the accession number GSE103499.

## References

[1] Bishop JM (1987). The molecular genetics of cancer. Science.

[2] Hahn WC (1999). Creation of human tumour cells with defined genetic elements. Nature.

[3] Guerrero S (2000). K-ras codon 12 mutation induces higher level of resistance to apoptosis and predisposition to anchorage-independent growth than codon 13 mutation or proto-oncogene overexpression. Cancer Res..

[4] Zhao B, Li L, Lei Q, Guan KL (2010). The Hippo-YAP pathway in organ size control and tumorigenesis: an updated version. Genes Dev..

[5] Zhao B, Tumaneng K, Guan KL (2011). The Hippo pathway in organ size control, tissue regeneration and stem cell self-renewal. Nat. Cell Biol..

[6] Sudol M (1994). Yes-associated protein (YAP65) is a proline-rich phosphoprotein that binds to the SH3 domain of the Yes proto-oncogene product. Oncogene.

[7] Huang J, Wu S, Barrera J, Matthews K, Pan D (2005). The Hippo signaling pathway coordinately regulates cell proliferation and apoptosis by inactivating Yorkie, the Drosophila Homolog of YAP. Cell.

[8] Dong J (2007). Elucidation of a universal size-control mechanism in Drosophila and mammals. Cell.

[9] Zhao B (2007). Inactivation of YAP oncoprotein by the Hippo pathway is involved in cell contact inhibition and tissue growth control. Genes Dev..

[10] Yu FX (2012). Regulation of the Hippo-YAP pathway by G-protein-coupled receptor signaling. Cell.

[11] Zhao B (2008). TEAD mediates YAP-dependent gene induction and growth control. Genes Dev..

[12] Yagi R, Chen LF, Shigesada K, Murakami Y, Ito Y (1999). A WW domain-containing yes-associated protein (YAP) is a novel transcriptional co-activator. EMBO J..

[13] Imajo M, Ebisuya M, Nishida E (2015). Dual role of YAP and TAZ in renewal of the intestinal epithelium. Nat. Cell Biol..

[14] Strano S (2005). The transcriptional coactivator Yes-associated protein drives p73 gene-target specificity in response to DNA Damage. Mol. Cell.

[15] Rosenbluh J (2012). beta-Catenin-driven cancers require a YAP1 transcriptional complex for survival and tumorigenesis. Cell.

[16] Zanconato F (2015). Genome-wide association between YAP/TAZ/TEAD and AP-1 at enhancers drives oncogenic growth. Nat. Cell Biol..

[17] Ye X, Deng Y, Lai ZC (2012). Akt is negatively regulated by Hippo signaling for growth inhibition in Drosophila. Dev. Biol..

[18] Tumaneng K (2012). YAP mediates crosstalk between the Hippo and PI(3)K-TOR pathways by suppressing PTEN via miR-29. Nat. Cell Biol..

[19] Manning BD, Toker A (2017). AKT/PKB Signaling: Navigating the Network. Cell.

[20] Linardopoulos S (1995). Deletion and altered regulation of p16INK4a and p15INK4b in undifferentiated mouse skin tumors. Cancer Res..

[21] Nishioka N (2009). The Hippo signaling pathway components Lats and Yap pattern Tead4 activity to distinguish mouse trophectoderm from inner cell mass. Dev. Cell.

[22] Pan D (2010). The hippo signaling pathway in development and cancer. Dev. Cell.

[23] Ota M, Sasaki H (2008). Mammalian Tead proteins regulate cell proliferation and contact inhibition as transcriptional mediators of Hippo signaling. Development.

[24] Vivanco, I. *et al*. A kinase-independent function of AKT promotes cancer cell survival. *Elife* **3** (2014). 10.7554/eLife.03751 PMC4337624 25551293

[25] Cusimano A (2015). Cytotoxic activity of the novel small molecule AKT inhibitor SC66 in hepatocellular carcinoma cells. Oncotarget.

[26] Andjelkovic M (1997). Role of translocation in the activation and function of protein kinase B. J. Biol. Chem..

[27] Blackwood EM, Eisenman RN (1991). Max: a helix-loop-helix zipper protein that forms a sequence-specific DNA-binding complex with Myc. Science.

[28] Huang MJ, Cheng YC, Liu CR, Lin S, Liu HE (2006). A small-molecule c-Myc inhibitor, 10058-F4, induces cell-cycle arrest, apoptosis, and myeloid differentiation of human acute myeloid leukemia. Exp. Hematol..

[29] Fossdal R (2004). A novel TEAD1 mutation is the causative allele in Sveinsson’s chorioretinal atrophy (helicoid peripapillary chorioretinal degeneration). Hum. Mol. Genet..

[30] Li Z (2010). Structural insights into the YAP and TEAD complex. Genes Dev..

[31] Kamijo T (1997). Tumor suppression at the mouse INK4a locus mediated by the alternative reading frame product p19ARF. Cell.

[32] Serrano M, Lin AW, McCurrach ME, Beach D, Lowe SW (1997). Oncogenic ras provokes premature cell senescence associated with accumulation of p53 and p16INK4a. Cell.

[33] Campisi J, d’Adda di Fagagna F (2007). Cellular senescence: when bad things happen to good cells. Nat. Rev. Mol. Cell Biol..

[34] Bellacosa A, Lazo PA, Bear SE, Tsichlis PN (1991). The rat leukocyte antigen MRC OX-44 is a member of a new family of cell surface proteins which appear to be involved in growth regulation. Mol. Cell. Biol..

[35] Coffer PJ, Woodgett JR (1991). Molecular cloning and characterisation of a novel putative protein-serine kinase related to the cAMP-dependent and protein kinase C families. Eur. J. Biochem..

[36] Jones PF, Jakubowicz T, Pitossi FJ, Maurer F, Hemmings BA (1991). Molecular cloning and identification of a serine/threonine protein kinase of the second-messenger subfamily. Proc Natl Acad Sci USA.

[37] Saxton RA, Sabatini DM (2017). mTOR Signaling in Growth, Metabolism, and Disease. Cell.

[38] Webb AE, Brunet A (2014). FOXO transcription factors: key regulators of cellular quality control. Trends Biochem. Sci..

[39] Mayer IA, Arteaga CL (2016). The PI3K/AKT Pathway as a Target for Cancer Treatment. Annu. Rev. Med..

[40] Croci O (2017). Transcriptional integration of mitogenic and mechanical signals by Myc and YAP. Genes Dev..

[41] Kitamura T (2003). Retrovirus-mediated gene transfer and expression cloning: powerful tools in functional genomics. Exp Hematol.

[42] Stewart SA (2003). Lentivirus-delivered stable gene silencing by RNAi in primary cells. RNA.

[43] Suzuki A (2016). Loss of MAX results in meiotic entry in mouse embryonic and germline stem cells. Nat Commun.

