# Supplementary material for: Transformation of normal cells by aberrant activation of YAP via cMyc with TEAD
Source: Sci Rep. 2019 Jul 29;9:10933. doi: 10.1038/s41598-019-47301-6 (PMC6662713; doi:10.1038/s41598-019-47301-6)
Supplement: Supplementary file 1 — supplemental Figures and Tables [file 41598_2019_47301_MOESM1_ESM.pdf]

Supplementary information

Transformation of normal cells by aberrant activation of YAP via cMyc with TEAD.

Masazumi Nishimoto, Kousuke Uranishi, Masamitsu N. Asaka, Ayumu Suzuki, Yosuke Mizuno, Masataka Hirasaki, Akihiko Okuda

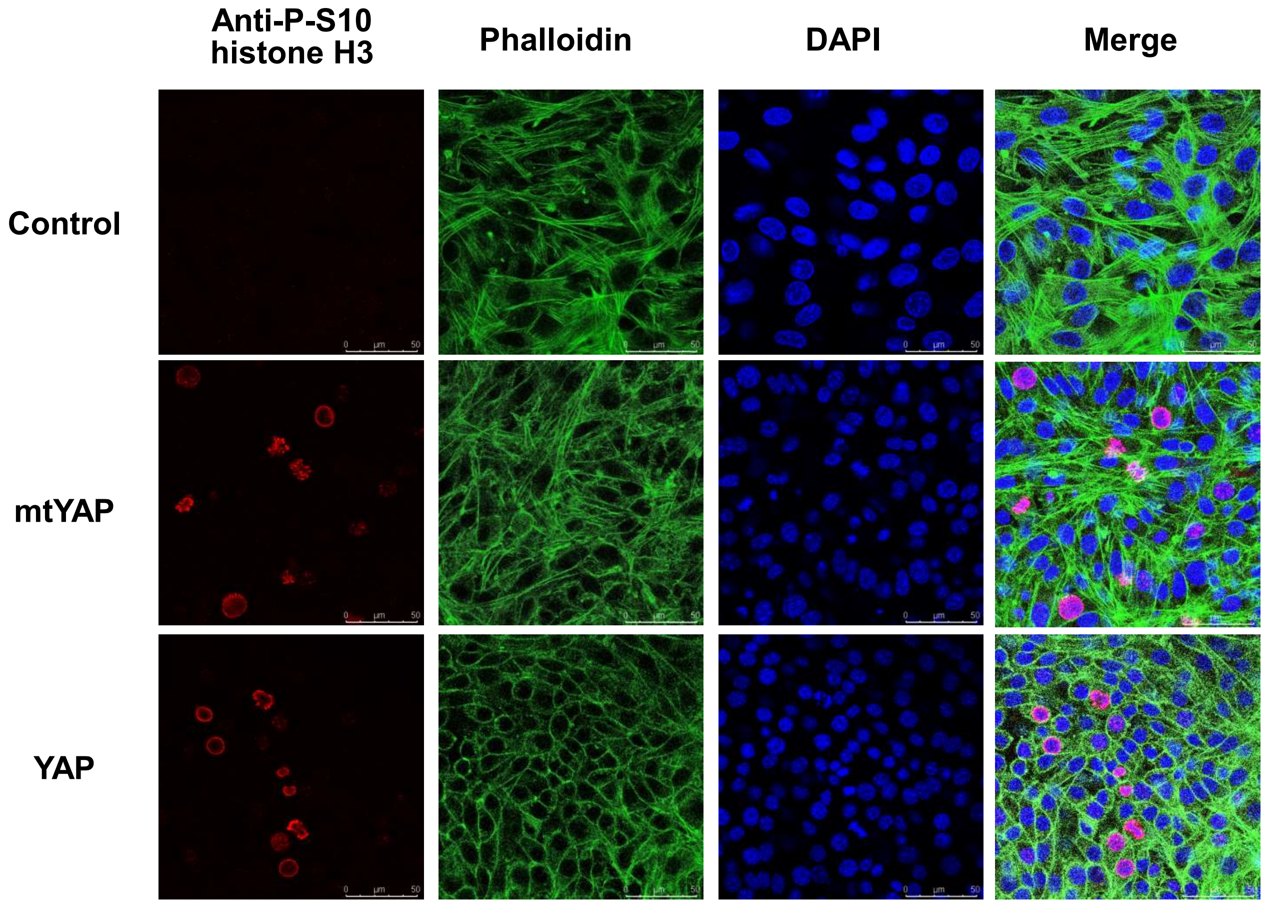

Supplemental Figure 1. Immunocytochemical analyses of phospho-histone H3 in control NIH3T3 cells and cells expressing YAP or mtYAP under confluent conditions. Two days after cells reached confluent conditions, cells were immunostained with anti-phospho-histone H3 (Ser 10). To confirm that cells were under confluent conditions, phallodin conjugated with iFour 488 dye was used to visualize actin filaments.

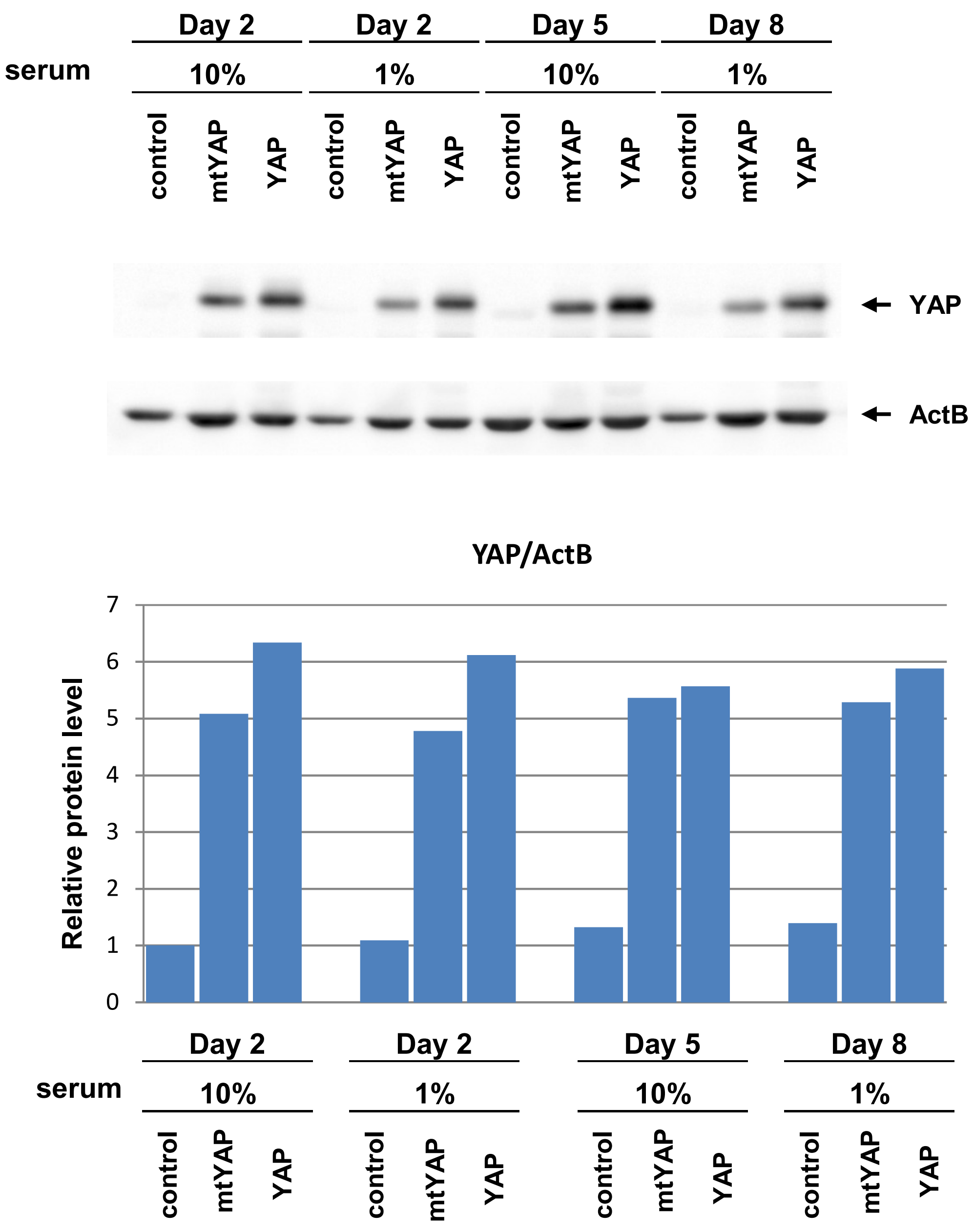

Supplemental Figure 2. Western blot analyses to examine the effect of cell density and serum levels on stabilization of YAP protein. Control NIH3T3 cells and cells overexpressing wild-type YAP or mtYAP were cultured with normal (10%) or low (1%) serum conditions. Whole cell extracts were prepared after two days (subconfluent condition) or five days (confluent condition) post transfer. YAP and ActB protein levels were examined by western blot analyses. Graph shows relative protein levels of YAP at various conditions normalized using ActB protein levels as an internal control. Protein level of YAP in control cells at day 2 is arbitrarily set to one.

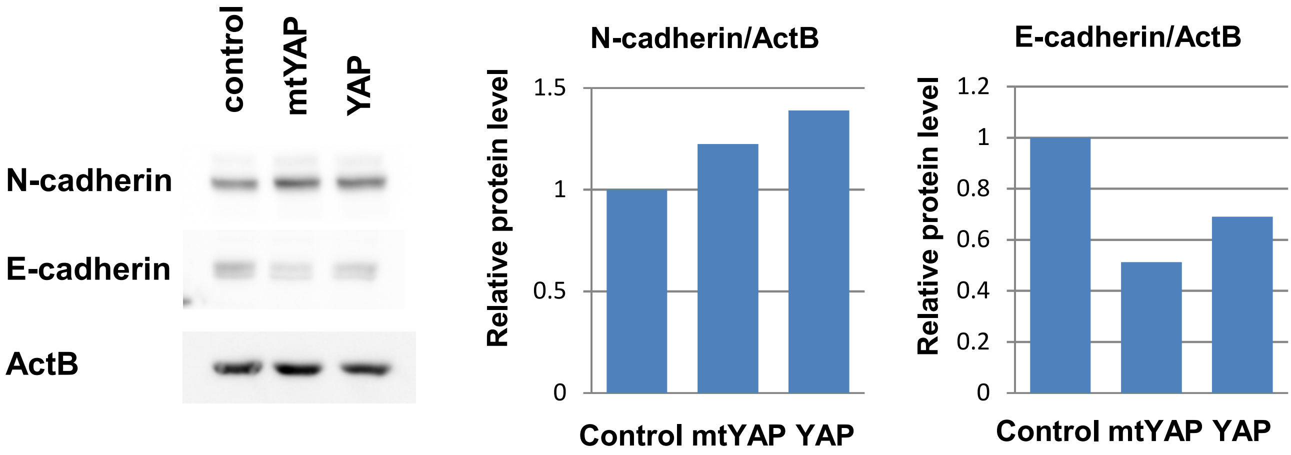

Supplemental Figure 3. Western blot analyses of N-cadherin and E-cadherin in control NIH3T3 cells and cells overexpressing wild-type YAP or mtYAP. Whole cell extracts were prepared from the indicated cells and protein levels of N-cadherin and E-cadherin proteins were assessed by western blot analyses. Graph shows the relative protein levels of N-cadherin and E-cadherin proteins normalized using ActB protein levels as an internal control.

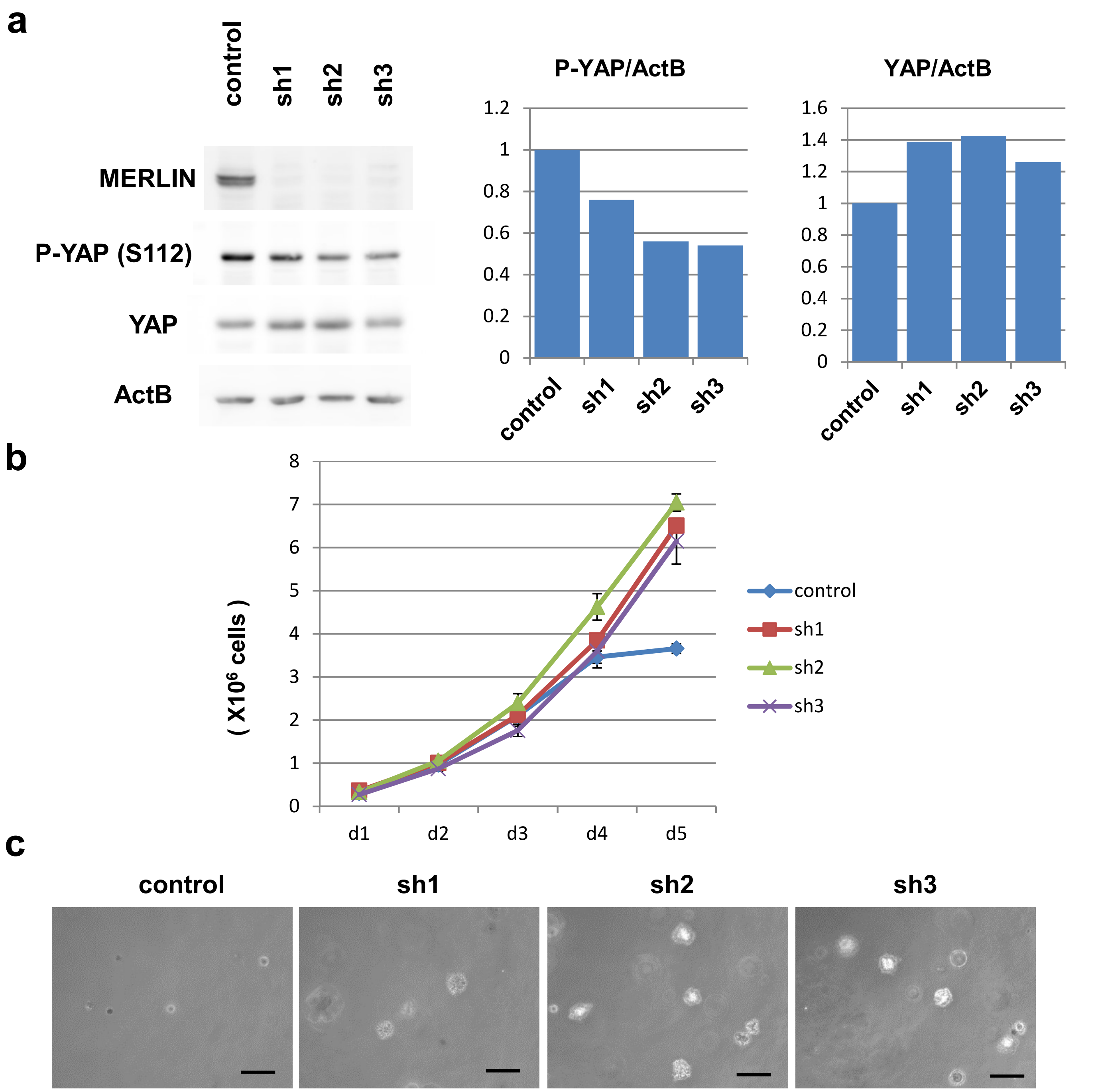

Supplemental Figure 4. The effect of deficiency of MERLIN on YAP activity. a. Western blot analyses to examine the effect of decrease in protein levels of MERLIN caused by *Nf2* gene knockdown on stability and phosphorylation of YAP. Three different lentivirus vectors (sh1, sh2 and sh3) for the knockdown were generated using oligonucleotides shown in Supplemental Table 2. Total and phosphorylated YAP protein levels were normalized using ActB protein levels as an internal control and the calculated data are shown as a bar graph. **b.** Growth curve of control NIH3T3 cells and cells with *Nf2* gene expression knock down with lentivirus vectors sh1, sh2 or sh3. **c.** Control NIH3T3 cells and cells subjected to *Nf2* gene knockdown by sh1, sh2 or sh3 were grown on agarose and colonies were observed by microscopy. The scale bar indicates 100 μm.

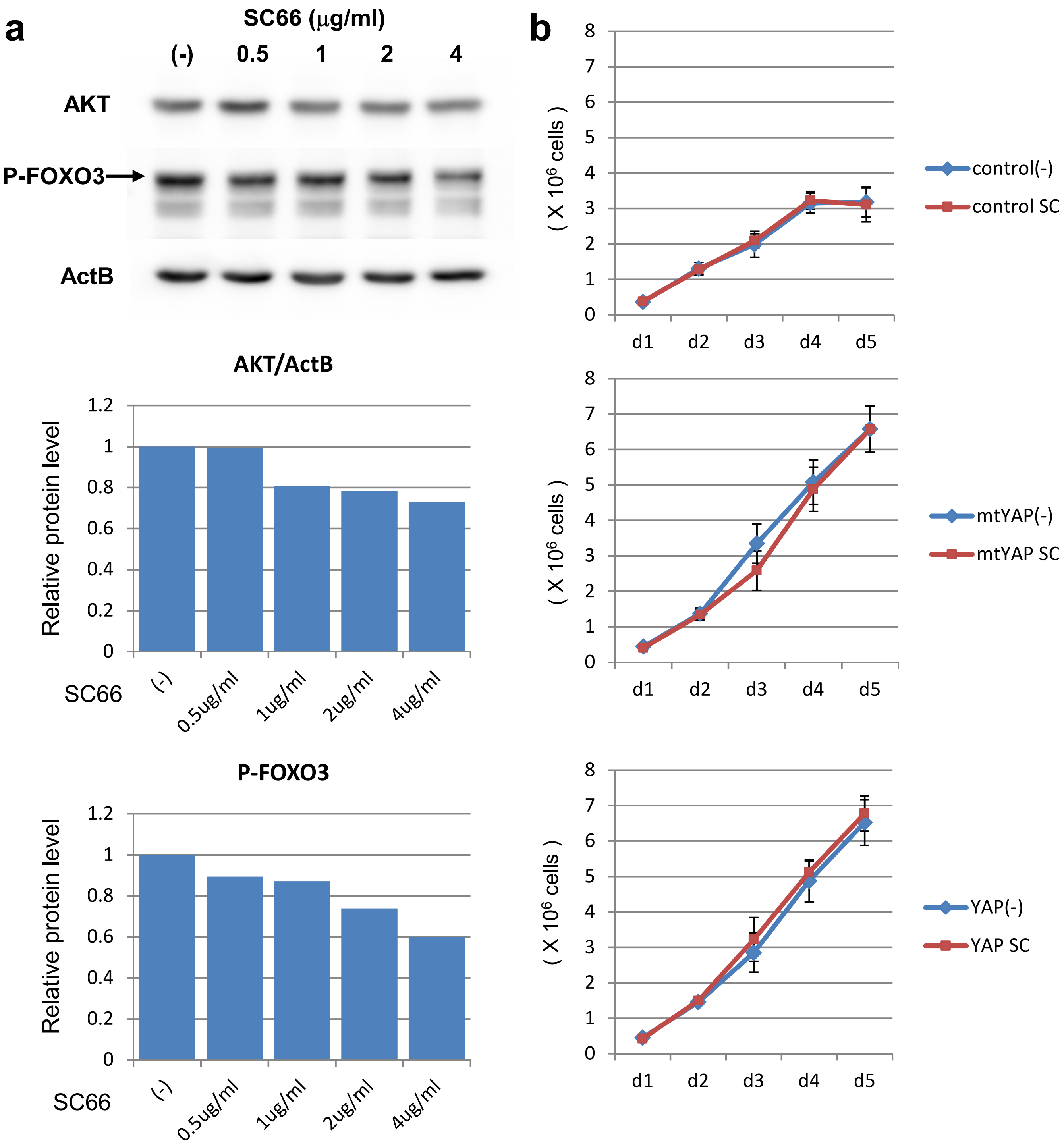

Supplemental Figure 5. The effect of SC66, an allosteric inhibitor against AKT, on the growth of controls NIH3T3 cells and cells overexpressing wild-type YAP or mtYAP. a. Western blot analyses of AKT and phosphorylated FOXO3 in NIH3T3 cells treated with SC66 at the indicated concentration. Protein levels of AKT and phosphorylated FOXO3 were normalized using ActB protein levels as an internal control and the calculated data are shown in graphs. **b.** Growth curve of control NIH3T3 cells and cells overexpressing wild-type YAP or mtYAP treated with or without SC66 (2 μg/ml).

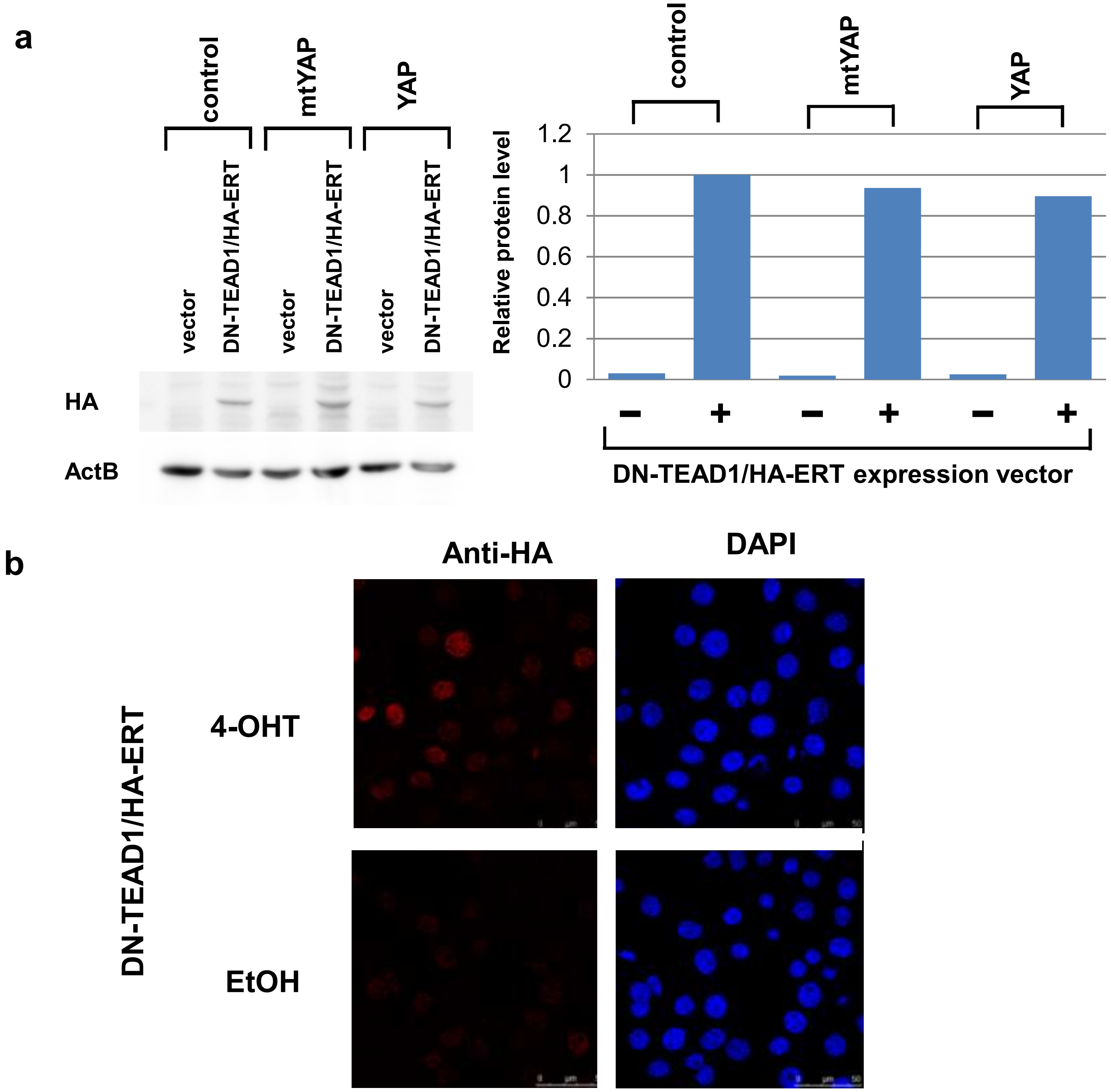

Supplemental Figure 6. Detection of dominant negative TEAD1/HA-ERT fusion protein and its nuclear localization by 4-OHT treatment. a. Whole cell extracts were prepared from control NIH3T3 cells and cells overexpressing wild-type YAP or mtYAP in which expression vector for DN-TEAD1/HA-ERT or empty vector was stably introduced. Lysates were used to detect DN-TEAD1/HA-ERT by western blot using anti-HA antibody. The protein levels of DN-TEAD1/HA-ERT were normalized using ActB protein levels as an internal control and calculated data are shown as a bar graph. **b.** NIH3T3 cells in which HA-tagged DN-TEAD1-ERT fusion protein was overexpressed were treated with 4-hydroxytamoxyfen (4-OHT) at 0.5 μM or only ethanol and then immunostained with anti-HA antibody.

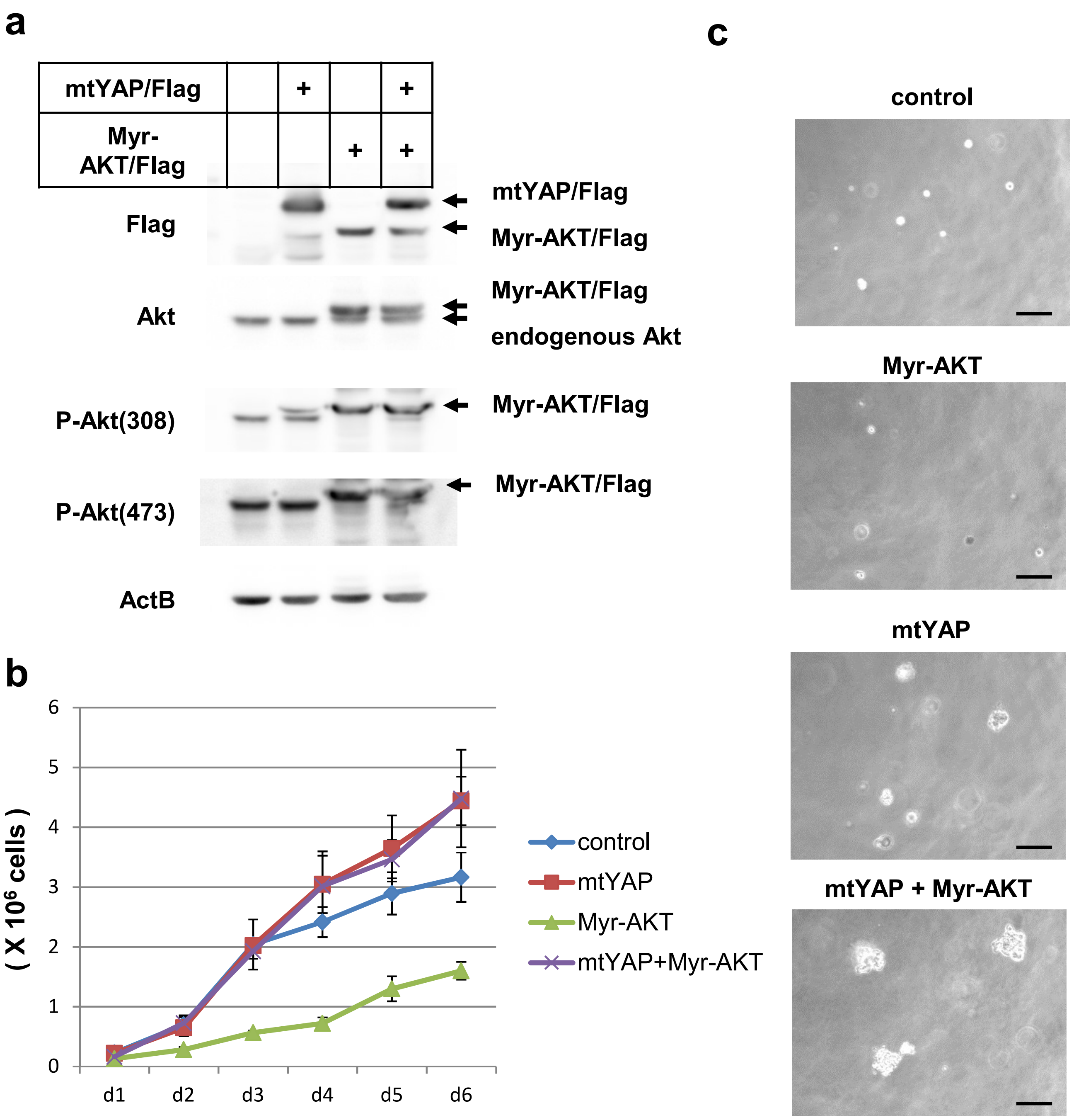

Supplemental Figure 7. Exploration of the possibility of functional cooperation between constitutively active AKT and YAP. a. Phosphorylation of Myr-AKT at threonine 308 and serine 473 of AKT were examined by western blot analyses using specific antibodies that recognize phosphorylated serine residues of AKT. **b.** Growth curve of control NIH3T3 cells and cells overexpressing mtYAP and/or Myr-AKT. **c.** Control NIH3T3 cells and cells overexpressing mtYAP and/or Myr-AKT were grown on agarose and anchorage independent growth was examined by monitoring colony formation under a microscope. The scale bar indicates 100 μm.

	Passage 7				Passage 32			
LT		+		+		+		+
mtYAP			+	+			+	+

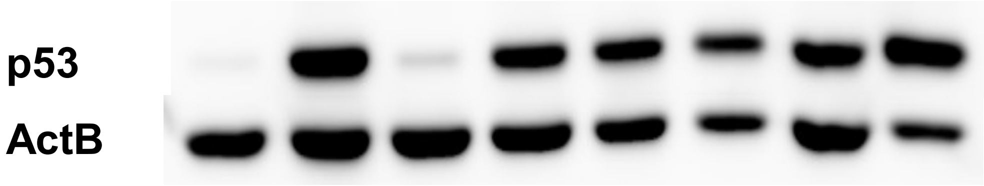

Supplemental Figure 8. Expression of p53 in control MEFs and cells overexpressing LT and/or mtYAP. Whole cell extracts were prepared from control MEFs and cells overexpressing LT and/or mtYAP at passage 7 and 32, and western blot analyses were performed for p53 levels. Control MEFs and cells overexpressing mtYAP alone showed accumulation of p53 protein at passage 32, but not at passage 7, confirming that these cells are non-immortalized cells and show senescence with relatively long culture. We also noted a prominent accumulation of p53 protein in MEFs overexpressing LT alone and those overexpressing LT and mtYAP at both short (passage 7) and long culture (passage 32). Since LT interacts with p53 and immortalizes normal cells, these results indicate that accumulation of p53 in LT-expressing MEFs at passage 7 does not indicate senescence, reflect the stabilizing effect of LT on p53.

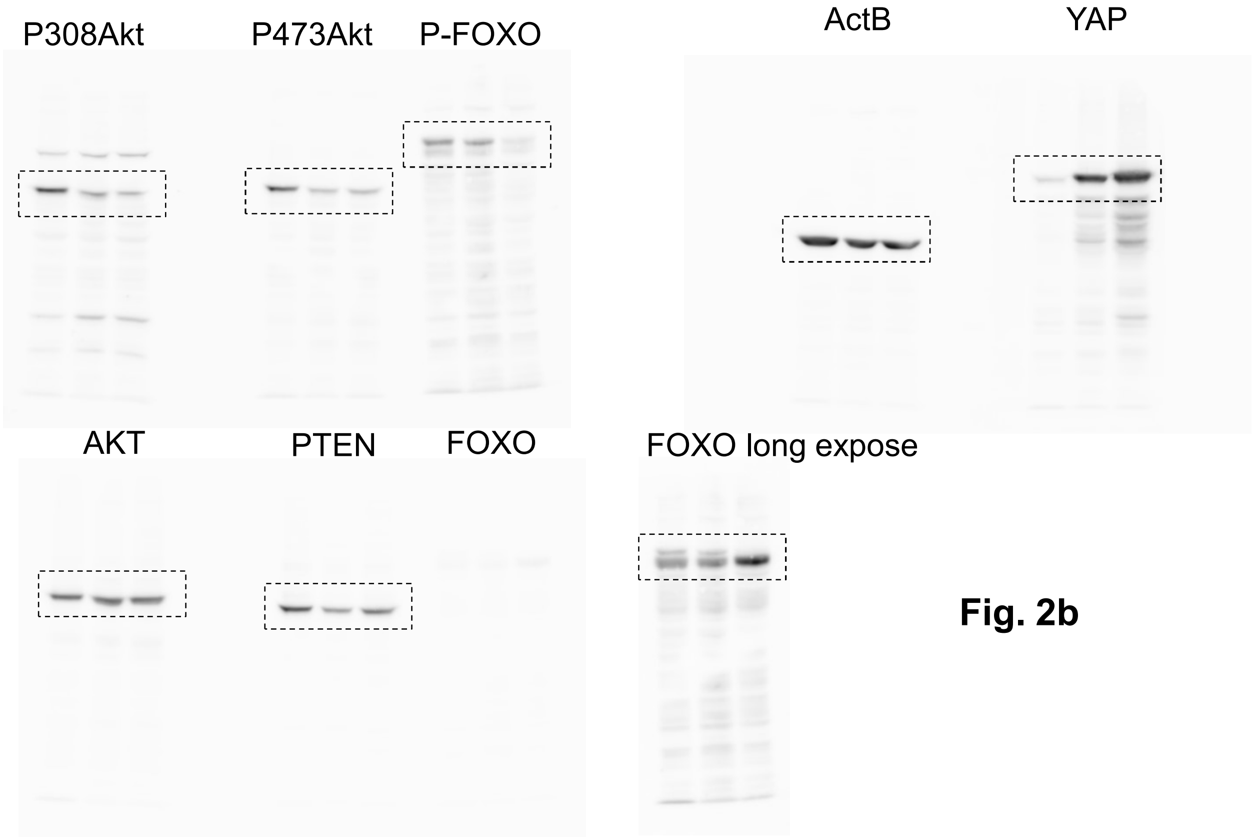
P308Akt
P473Akt
P-FOXO
ActB
YAP
AKT
PTEN
FOXO
FOXO long expose

Fig. 2b

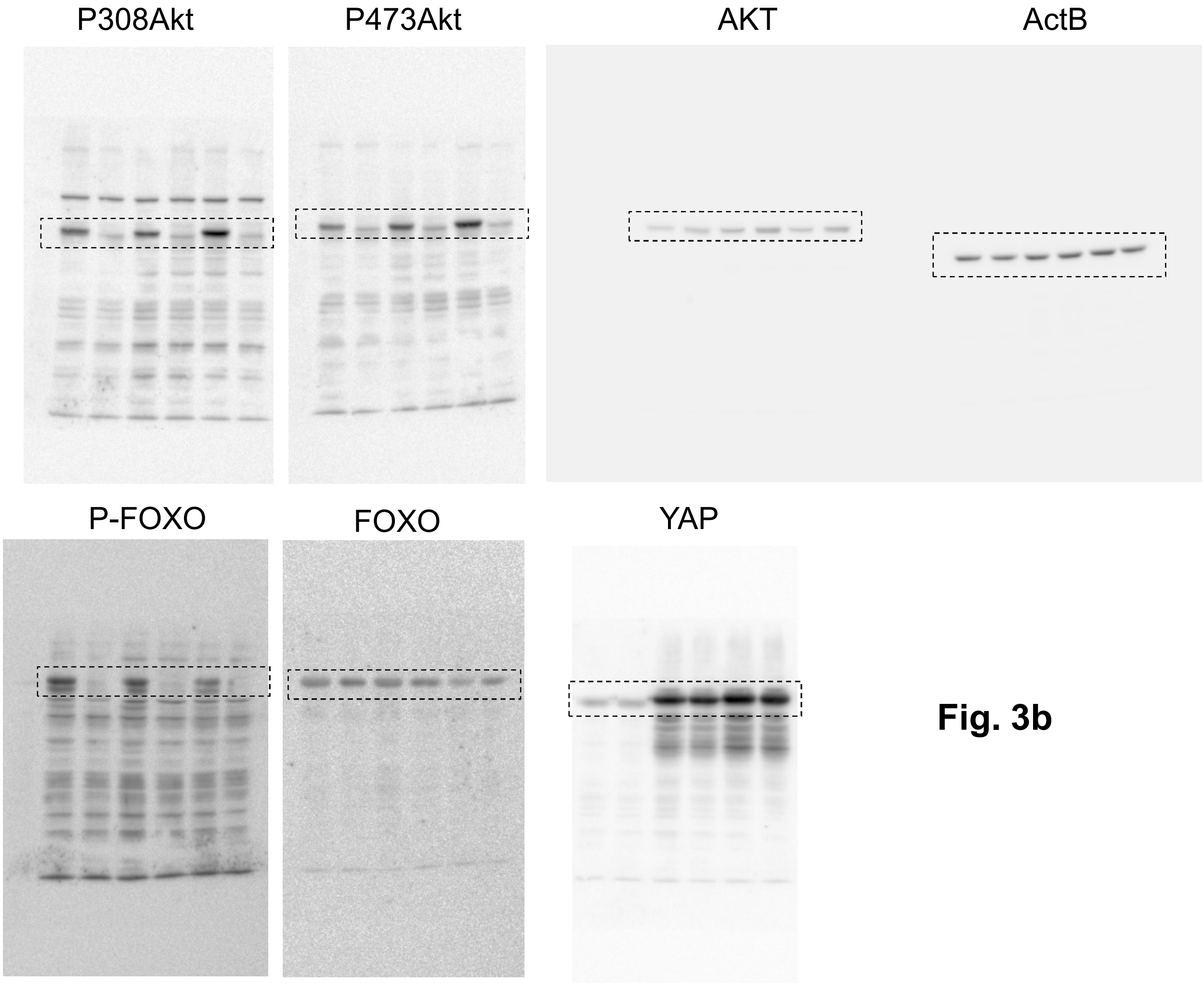
P308Akt
P473Akt
AKT
ActB
P-FOXO
FOXO
YAP

Fig. 3b

Fig. 7c

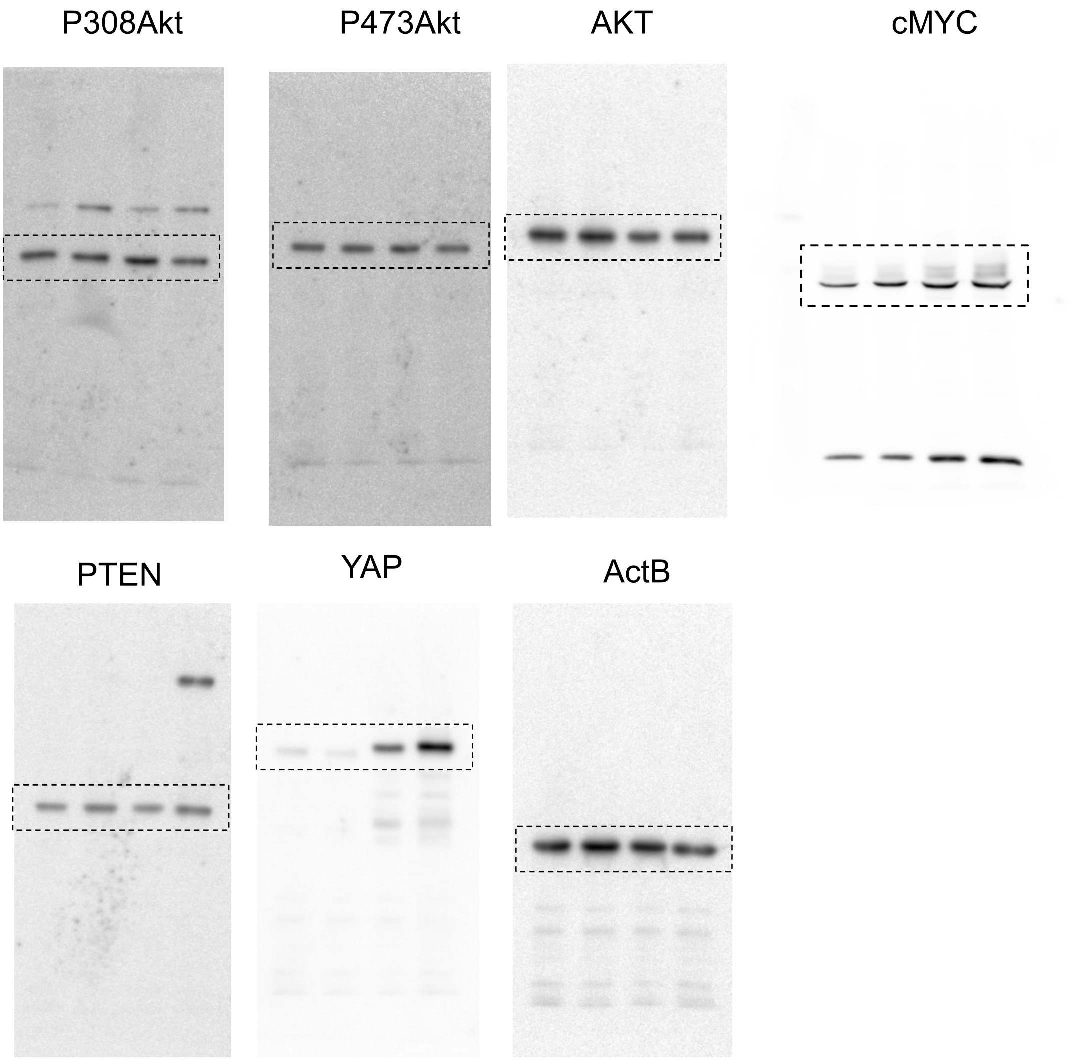

Fig S1

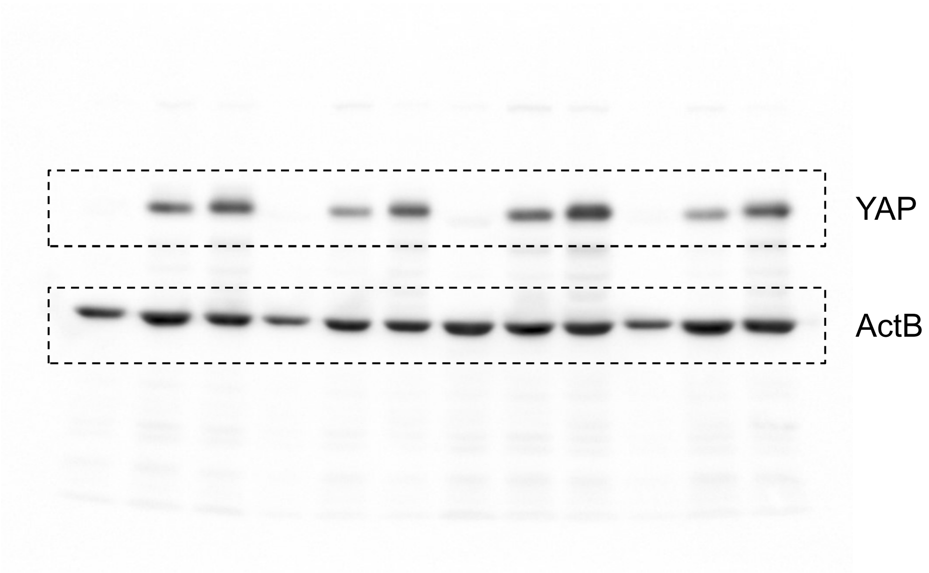

Fig S3

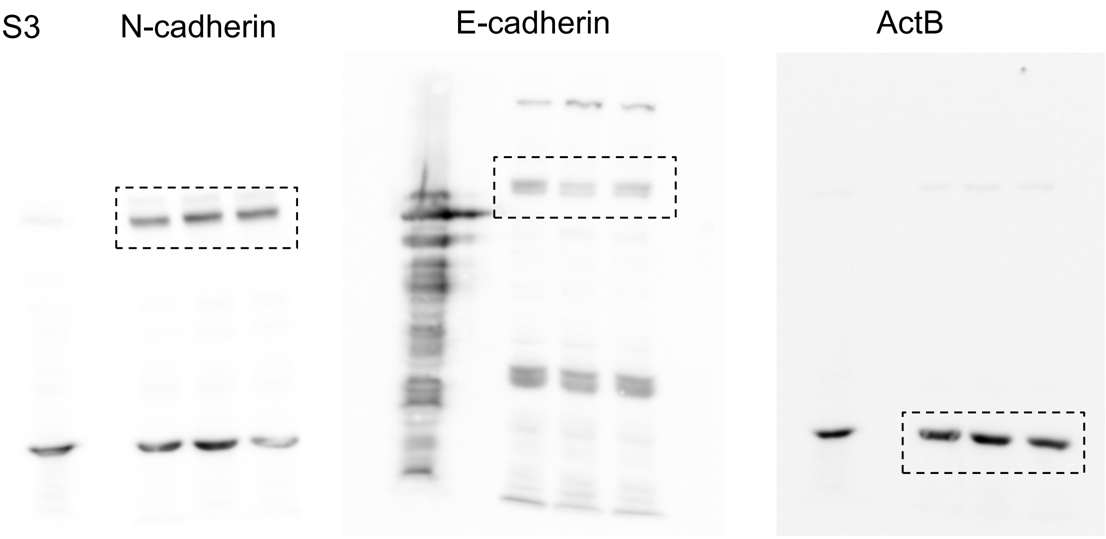

Fig S4

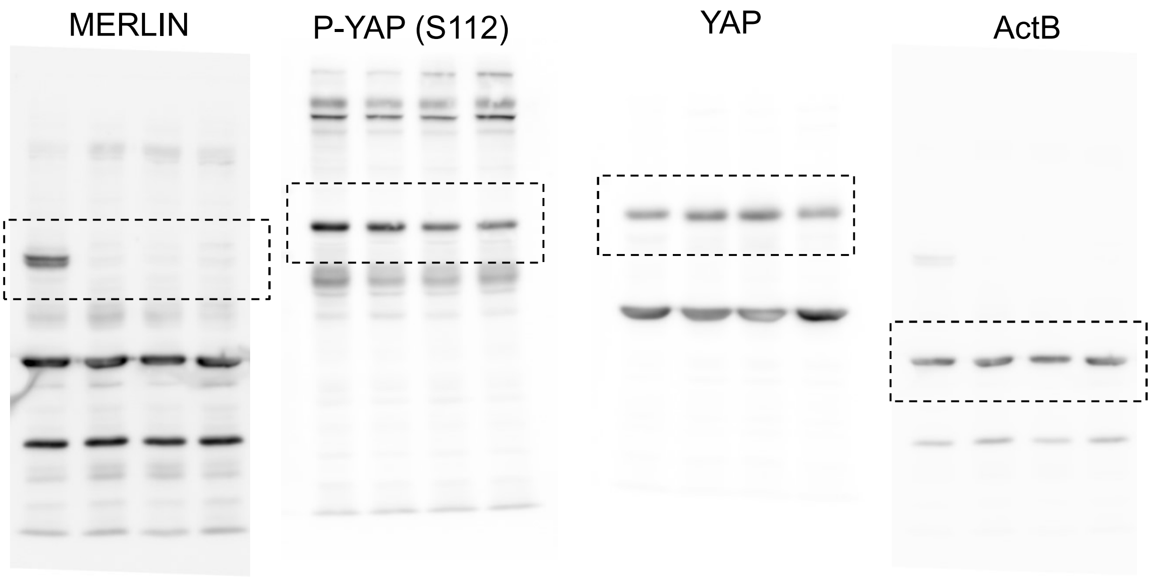

Fig S5

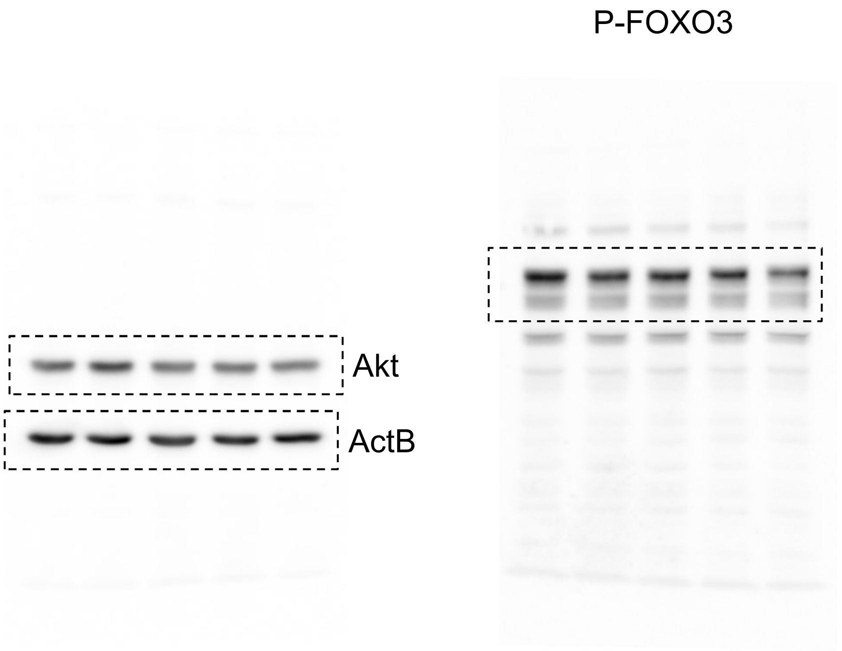

Fig. S6

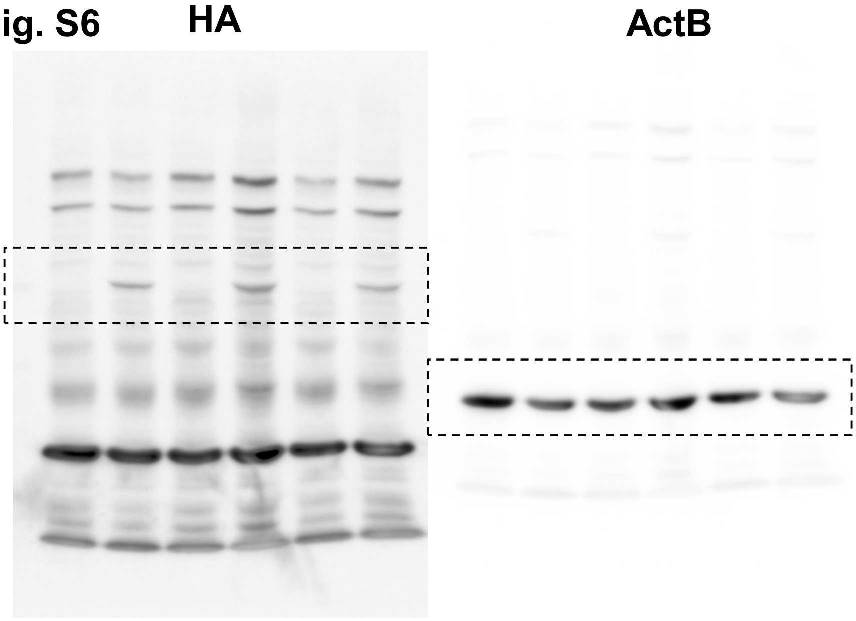

Fig. S7

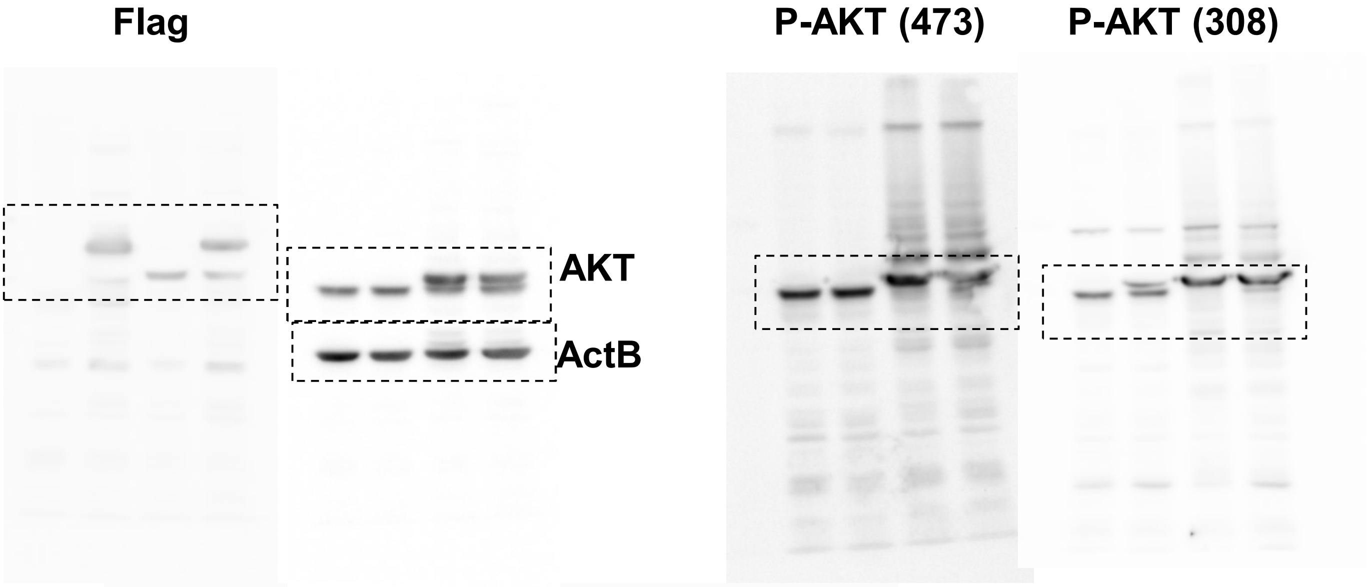

Fig. S8

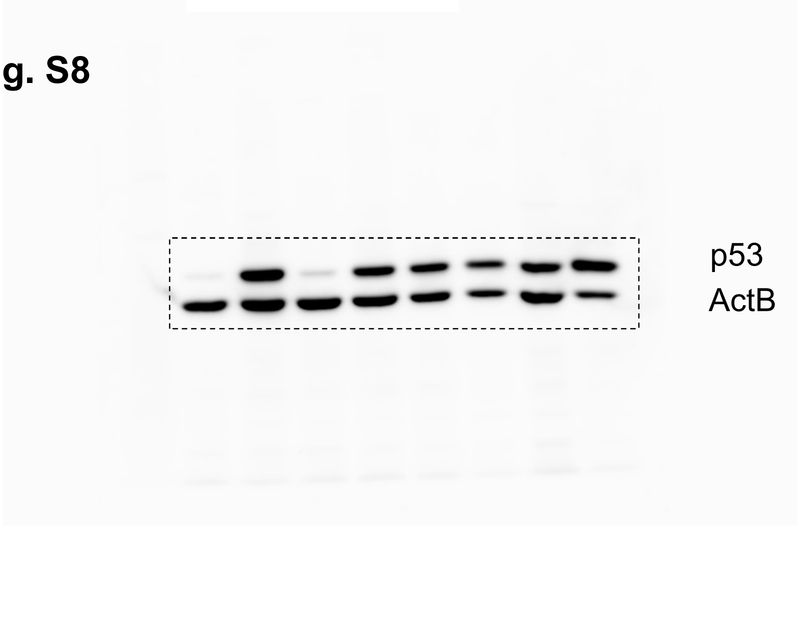

Supplementary Table 1. Primers used for qRT-PCR

Target RNA	Forward Primer	Revers Primer	Product Size (bp)
Ctgf	TGACCTGGAGGAAAACATTAAGA	AGCCCTGTATGTCTTCACACTG	112
Serpine1	AGGATCGAGGTAAACGAGAGC	GCGGGCTGAGATGACAAA	66
cMyc	TTTGTCTATTTGGGGACAGTGTT	CATCGTCGTGGCTGTCTG	128
Ccdn2	GGCCAAGATCACCCACACT	ATGCTGCTCTTGACGGAACT	103
β–actin	CTAAGGCCAACCGTGAAAAG	ACCAGAGGCATACAGGGACA	104

Supplementary Table 2. Design of shRNA expression vector for Merlin knockdown.

	sequence
Merlin sh1	GGACAAGAAGGTGTTGGATCATTCAAGAGATGATCCAACACCTTCTTGTCCTTTTTT
Merlin sh2	GCTAGAAAGCAGATGGAAAGGTTCAAGAGACCTTTCCATCTGCTTTCTAGCTTTTTT
Merlin sh3	GCGACTTTCCATGGAGATAGATTCAAGAGATCTATCTCCATGGAAAGTCGCTTTTTT

Supplementary Table 3. Antibodies used for immunoblotting and immunofluorescence

**Target protein**	**Supplier**	**Dilution Immuno-blotting**	**Dilution Immuno-fluorescence**
Akt	#9272-Cell Signaling Technology	1:1000	
Phospho-Akt (Ser473)	#9271-Cell Signaling Technology	1:1000	
Phospho-Akt (Thr308)	#2965-Cell Signaling Technology	1:1000	
FoxO1	#2880-Cell Signaling Technology	1:1000	
Phospho-FoxO1 (Thr24) PhosphoFoxO3a (Thr32)	#9464-Cell Signaling Technology	1:1000	
PTEN	#9559-Cell Signaling Technology	1:1000	
Yap	#12395-Cell Signaling Technology	1:1000	1:400
p53	#2524-Cell Signaling Technology	1:1000	
cMyc	#5605-Cell Signaling Technology	1:1000	
Phospho-Histone H3 (Ser10)	#9701-Cell Signaling Technology		1:400
E-cadherin	#3195-Cell Signaling Technology	1:1000	
N-cadherin	#14215-Cell Signaling Technology	1:1000	
Merlin	#12888-Cell Signaling Technology	1:1000	
β–actin	sc47778-SANTACRUZ Technology	1:1000	
HA-tag	H3663-Sigma-Aldrich		1:1000
Alexa-594 conjugated anti-mouse IgG	A11005-Thermo Fisher Scientific		1:400
HRP conjugated anti-mouse IgG	#7076-Cell Signaling Technology	1:3000	
HRP conjugated anti-rabbit IgG	#7074-Cell Signaling Technology	1:3000	

Supplemental Table 4

	512 common genes	mtYAP / control (log2)	YAP / control (log2)
1	Car6	6.84	5.73
2	Fgf21; LOC1052424	5.85	2.21
3	Cth	5.55	3.78
4	Cdsn	4.97	3.98
5	Paqr3	4.79	3.75
6	Stbd1	4.54	1.88
7	Plscr2	4.4	1.53
8	Ankrd1	4.23	2.8
9	Msln	4.18	3.6
10	Ppp1r15a	3.88	2.3
11	Cyp3a13	3.66	3.2
12	Sema3c	3.635	2.375
13	Gadd45a	3.58	2.3
14	Trib3	3.57	2.85
15	Adam22	3.475	2.505
16	Steap1	3.465	2.295
17	Gm38403	3.44	2.98
18	Ero1l	3.4	2.413333333
19	Ptgs2	3.365	1.9
20	Dppa2	3.35	1.445
21	Ptx3	3.34	1.65
22	Angptl6	3.31	2.74
23	Slc6a15	3.26	2.49
24	Hbegf	3.24	1.12
25	5430416N02R	3.23	1.67
26	Rnd1	3.23	1.36
27	Aldh1a1	3.22	2
28	Rab39b	3.21	1.805
29	Tmem182	3.21	2.31
30	Atf3	3.16	1.65
31	Tmem45a	3.14	2.86
32	Serpinb9g	3.12	3.14
33	Psph	3.1	1.97
34	**cMyc**	2.96	1.91
35	Anxa3	2.96	1.86
36	Chka	2.94	1.86
37	Plagl1	2.93	0.783333333
38	Gm13152	2.86	1.94
39	Serpinb9b	2.81	1.245
40	Cyb5r1	2.77	1.98
41	Chac1	2.74	1.35
42	Tenm4	2.736666667	1.453333333
43	Gja1	2.701666667	1.771666667
44	Edn1	2.68	0.99
45	Slc7a5	2.66	1.88
46	Pkia	2.615	1.205
47	Rras2	2.58	1.21
48	Snhg1	2.57	1.6

49	Chrnb1	2.57	1.643333333
50	Hectd2	2.54	1.835
51	Npr3	2.54	1.036666667
52	Sh2d6	2.5	1.41
53	Dkc1	2.476666667	1.396666667
54	4930431P03R	2.47	1.08
55	Fibin	2.47	1.01
56	Mcm2	2.46	0.995
57	Ect2	2.41	1.32
58	Odc1	2.395	1.24
59	Slc25a33	2.38	1.56
60	Mtm1	2.373333333	1.363333333
61	Nrcam	2.36	1.44
62	Isoc1	2.35	1.5325
63	Zwilch	2.33	1.32
64	Mthfd2	2.325	1.515
65	Brinp3	2.32	2.05
66	Tmcc3	2.312	0.73
67	Flrt2	2.296666667	1.83
68	Styk1	2.27	1.25
69	E2f7	2.26	0.94
70	Fads3	2.26	1.473333333
71	Ms4a4d	2.26	1.69
72	Rad51	2.26	0.72
73	Ak5	2.245	0.665
74	Ncapg	2.24	1.34
75	Tfec	2.24	2.65
76	Cpox	2.225	1.11
77	Cdca7	2.2	1.27
78	Depdc1a	2.2	1.24
79	Gch1	2.2	0.695
80	Wars	2.2	1.2325
81	Tmem47	2.2	1.91
82	Cks2	2.19	0.84
83	Alcam	2.16	1.7175
84	Syce2	2.14	1.01
85	Cdca8	2.133333333	1.293333
86	Epha2	2.13	0.68
87	Pbk	2.13	1.01
88	Nop58	2.11	1.16
89	Itga6	2.11	1.825
90	Cenpq	2.09	0.69
91	Murc	2.09	1.38
92	Mpp6	2.085	1.555
93	Htra1	2.07	1.78
94	Oxr1	2.07	0.93
95	Prc1	2.065	1.32
96	Nop56	2.06	1.11
97	Ereg	2.04	0.8
98	Rrp12	2.04	1.02
99	Nrg1	2.03	1.54
100	Gm10058;<br>Gm10096;	2.03	2.02
101	Sdpr	2.023333333	1.433333333

102	Anln	2.02	1.1
103	Diaph3	2.02	1
104	Spc24	2.02	1.13
105	D8Ertd56e	2.02	0.68
106	BC010981	2.02	0.91
107	Ube2c	2.02	1.36
108	Asns	2.015	1.575
109	Ldlrad3	2.01	1.73
110	Lonp1	2	1.56
111	Reep6	2	0.86
112	9130008F23R	2	1.28
113	Shmt2	1.9925	1.195
114	Ccnb1; Gm5593	1.99	1.21
115	**Mycn**	1.99	2.196666667
116	Lyar	1.98	1.05
117	Clcn3	1.97	1.295
118	Pard6b	1.955	0.71
119	Rin1	1.94	1.63
120	Wdr43	1.935	1.005
121	Slc7a11	1.93	1.51
122	Serpini1	1.93	1.296666667
123	Specc1	1.926666667	0.77
124	Wnt9a	1.925	1.055
125	Snhg1;	1.92	1.05
126	Rad51ap1	1.9175	0.75
127	Abcc4	1.91	2.03
128	Snhg12	1.91	0.96
129	2010204K13R	1.91	1.22
130	Agpat9	1.9	1.57
131	Trip13	1.9	0.946666667
132	Pla2g12a	1.9	0.89
133	Klra1; Klra22	1.89	2.11
134	Kntc1	1.89	0.92
135	4921524J17Ri	1.89	0.92
136	Acot2	1.89	1.37
137	Ddit3	1.89	1.1
138	Slc7a1	1.886666667	1.183333333
139	Cenpe	1.885	0.79
140	Pole	1.88	0.77
141	Serpinb6b	1.88	1.97
142	Bcat1	1.866666667	1.526666667
143	BB557941	1.86	0.7
144	Cyp3a41a; Cyp3a41b	1.86	0.72
145	Prps1	1.86	1.26
146	Stil	1.85	0.96
147	Fam195a	1.85	0.97
148	Tmem185b	1.85	2.17
149	P2rx3	1.833333333	2.253333333
150	Rbpj	1.833333333	0.84
151	Avil	1.83	1.3
152	Dbt	1.83	1.12
153	Rfc4	1.82	1.04

154	Cenpi	1.82	1.115
155	Iars	1.815	1.34
156	Ndc80	1.81	1.22
157	Zbtb18	1.81	1.13
158	Dut	1.805	1.53
159	Serpinb1a	1.8	2.73
160	Slc25a40	1.8	0.94
161	Aldh1l2	1.8	1.38
162	BC023969	1.79	0.9
163	Cep55	1.79	0.795
164	Eda2r	1.79	1.47
165	Plk4	1.786666667	1.003333333
166	Sgf29	1.786666667	1.2
167	Nupr1	1.78	0.74
168	Pla2g4a	1.78	1.14
169	Rpp40	1.775	1.3
170	Timm8a1	1.775	0.92
171	AI662270	1.77	1.14
172	Fam107b	1.766666667	0.896666667
173	Fam60a; Gm41465; LOC1026367	1.76	0.96
174	Lars	1.76	1.22
175	Taf7l	1.75	1.52
176	Usp17la	1.75	1.05
177	Srm	1.75	0.88
178	Dcbld2	1.746666667	1.02
179	Tbpl1	1.745	1.175
180	Loxl4	1.745	1.015
181	Hivep1	1.74	0.99
182	Mcm5	1.736666667	0.776666667
183	Sgcd	1.733333333	1.24
184	Gpt2	1.7325	1.215
185	Dnph1	1.73	1.09
186	Atad3a	1.72	0.8975
187	Rarb	1.72	1.44
188	Serpine1	1.72	1.08
189	Mfsd9	1.71	1.85
190	Gprc5a	1.71	1.16
191	Nars	1.705	1.115
192	Melk	1.7	1.06
193	Spdl1	1.7	0.94
194	Ltv1	1.69	0.7
195	Emx2	1.69	0.82
196	Gm41724	1.69	0.68
197	Fabp4	1.685	1.395
198	Noc2l	1.68	1.45
199	Leprotl1	1.675	0.75
200	AI506816	1.66	0.95
201	Dis3	1.66	0.75
202	Dctd	1.65	0.85
203	Spag5	1.65	1.093333333
204	Wdr74	1.65	0.85
205	Ddx39	1.646	0.898

206	Rrp15	1.64	0.94
207	Aurkb	1.64	1.075
208	Nup43	1.64	0.855
209	Erlin1	1.635	1.02
210	Gnpnat1	1.63	0.826666667
211	Morc4	1.63	1.05
212	Lamp3; Ppid	1.63	1.08
213	Mybbp1a	1.63	1.045
214	Nhp2	1.63	0.845
215	Ddias	1.625	0.765
216	Snrpa1	1.613333333	0.85
217	Kpna2	1.61	0.71
218	Plaur	1.61	0.69
219	Tomm70a	1.6075	0.8975
220	Glce	1.6	1.35
221	Pvrl2	1.6	0.98
222	Ly75	1.6	1.03
223	Mthfd1l	1.6	1.71
224	Gtpbp4	1.596666667	1.05
225	Ccser1	1.595	0.87
226	Cenpw	1.59	0.68
227	Hist1h1e	1.59	0.94
228	Taf15	1.58	1.02
229	Uchl3; Uchl4	1.58	0.82
230	Erc2	1.58	1.49
231	Mreg	1.58	0.66
232	Ruvbl2	1.58	1.05
233	Zdhhc2	1.5675	1.33
234	Ndrg1	1.566	0.942
235	Eif4ebp1	1.563333333	0.86
236	Pcyt2	1.56	1.53
237	Eef1e1	1.56	0.71
238	Ifrd2	1.55	0.85
239	Kif22	1.546666667	1.163333333
240	Strbp	1.54	1.14
241	Dbf4	1.53	0.9
242	B3galnt2	1.526666667	1.303333333
243	Dgat2	1.525	0.665
244	Tjp2	1.5225	1.04
245	Zfp930	1.515	0.89
246	Pa2g4	1.514	0.924
247	Sap30	1.51	0.89
248	Slc4a4	1.508	0.818
249	Ddx21	1.505	1.05
250	Tceal	1.505	1
251	Pkp2	1.505	0.9725
252	Cdca7l	1.5	0.68
253	Jade3	1.5	1.04
254	Hells	1.496666667	0.753333333
255	Sgol1	1.495	0.735
256	Nop14	1.49	0.82
257	Yars	1.49	0.75
258	Arhgap11a	1.49	0.79
259	Snhg17	1.49	0.67

260	Klk8	1.48	1.58
261	Pvt1	1.473333333	0.723333333
262	Nsun2	1.47	0.83
263	Pold2	1.47	0.93
264	Dppa4	1.47	0.93
265	Slc16a6	1.47	0.9
266	Wwc1	1.47	0.6675
267	Pdgfc	1.465	1.125
268	Nomo1	1.465	0.755
269	Bora	1.46	0.9
270	Phgdh	1.46	0.9925
271	Insl6	1.45	1.4
272	Timm9	1.45	0.68
273	Mcm8	1.45	0.69
274	Tbc1d31	1.446666667	1.136666667
275	Anxa8	1.445	1.83
276	Tspan9	1.44	0.965
277	Klf4	1.44	1.33
278	Dusp9	1.436666667	1.046666667
279	Adat2	1.43	1.2
280	Gtf2f2	1.43	0.85
281	Igf2	1.43	1.475
282	Ascc2	1.425	0.87
283	Zfpm2	1.425	1.055
284	Cenpm	1.42	0.69
285	Pus7	1.42	0.75
286	Tex30	1.42	0.77
287	Cmss1	1.415	1.195
288	Ctps	1.41	0.74
289	Eno3	1.41	1.17
290	Plekha1	1.41	1.07
291	Prps1; Prps1l3	1.41	0.97
292	Farsb	1.41	0.6875
293	Nol10	1.41	0.79
294	Steap2	1.408	1.068
295	Psat1	1.4	1.15
296	Gmnn	1.4	0.98
297	Slc1a5	1.395	1.015
298	Zfp958	1.39	0.91
299	Wisp1	1.39	0.995
300	Clpb	1.385	1.34
301	Fam110c	1.38	1.06
302	Cdc42se2	1.38	0.86
303	Ncaph	1.38	0.85
304	Nup85	1.38	0.765
305	Fabp5	1.38	0.81
306	Gnl3	1.38	0.75
307	Aldh18a1	1.378	0.896
308	Hmmr	1.372	0.966
309	Immp2l	1.37	0.8
310	Pola1	1.37	0.84
311	Rhox5	1.37	1.23
312	2700029M09 Rik	1.37	0.67

313	Snhg5	1.365	0.77
314	Rpp30	1.36	0.8
315	Lin9	1.36	0.68
316	Palb2	1.36	0.93
317	Rif1	1.356	0.818
318	LOC1026426 19; Prdx6	1.355	0.865
319	Mbnl3	1.353333333	0.85
320	Espl1	1.34	0.97
321	Fam111a	1.34	1.04
322	Fst	1.34	0.87
323	Tacc3	1.34	0.786666667
324	Cdc25c	1.335	0.85
325	Ebna1bp2	1.335	0.815
326	Arhgap33	1.33	1.07
327	Birc5	1.33	0.69
328	Zfp593	1.33	0.915
329	Ept1	1.3275	0.7975
330	Nudcd1	1.325	0.965
331	Ftsj3	1.32	0.67
332	Klra18	1.32	0.87
333	Metrnl	1.32	1.33
334	Acer3	1.315	0.7475
335	Plod2	1.315	1.165
336	Ttk	1.315	0.755
337	Arhgap5	1.31	0.6675
338	Gstcd	1.31	0.67
339	Siglecg	1.31	1.47
340	Arhgap29	1.306666667	0.803333333
341	Pdzd8	1.305	0.875
342	Abcb1b	1.305	0.9
343	Casc5	1.305	0.895
344	Naa50	1.3	0.82
345	Hacd4	1.3	1.15
346	Cacybp	1.3	0.7
347	Rsl1d1	1.3	0.84
348	3110043O21R	1.29	0.68
349	Saa3	1.29	0.78
350	Tmem194	1.29	0.76
351	Llph	1.29	0.73
352	Cenpa	1.285	0.79
353	Nipal1	1.285	0.86
354	Gemin2	1.28	1.04
355	Htatip2	1.28	0.91
356	Mnd1	1.28	1.55
357	Kif20a	1.28	1.68
358	Polr1e	1.275	1.005
359	Cdt1	1.273333333	0.69333
360	Slc25a37	1.27	0.75
361	Snrpf	1.27	0.95
362	Cd180	1.26	0.96
363	Fam171b	1.26	0.945
364	Ifrd1	1.26	0.66
365	Ovgp1	1.26	0.82

366	Ckap2	1.26	1.12
367	Eif3j1; Eif3j2	1.253333333	0.78
368	Aire	1.25	0.77
369	Lrrc75a	1.25	0.98
370	Nutf2; Nutf2-ps2	1.25	0.65
371	Rap1gap2	1.25	0.88
372	Slc6a9	1.25	0.88
373	Tagln2	1.25	0.843333333
374	BC055324	1.245	0.82
375	Cdc20	1.243333333	0.926667
376	Pcgf6	1.24	0.656666667
377	Acot10; Acot9	1.24	0.98
378	Gm4070;	1.24	0.96
379	Kifc1	1.24	0.68
380	Tdrkh	1.24	0.75
381	Nob1	1.24	1.03
382	Gm30289; Larp1b	1.235	0.925
383	Fermt3	1.233333333	0.653333333
384	Serpinb1b	1.23	1.92
385	Gm4371	1.23	1
386	Prpf3	1.23	0.72
387	Myom2	1.2275	0.6775
388	Hprt	1.22	0.84
389	Tdp2	1.22	0.82
390	2200002D01R	1.22	0.92
391	Ppan	1.22	0.76
392	Rcc1	1.22	0.86
393	Tars	1.22	0.7
394	Prss23	1.216666667	0.966666667
395	2700099C18R	1.21	0.77
396	Me3	1.21	1.09
397	Med30	1.21	0.67
398	Emp2	1.2	0.93
399	Pnkp	1.2	0.86
400	Tmem171	1.2	0.7
401	Ahcy;	1.2	1.13
402	Gars	1.2	1.04
403	Hmgn5	1.2	0.73
404	Prr11	1.2	1.145
405	Socs6	1.193333333	0.8
406	Foxp1	1.19	0.728571429
407	Mtbp	1.19	0.71
408	Prim1	1.19	0.79
409	Rdh9	1.19	1.11
410	Prmt3	1.19	0.66
411	Ybx3	1.19	0.94
412	Bri3bp	1.185	0.855
413	Ipo5	1.185	0.95
414	Knstrn	1.185	0.91
415	Mrpl32	1.18	0.71
416	Rassf7	1.18	1.08
417	Mir155hg	1.18	1.63

418	4930432K21R	1.18	0.67
419	Abcc1	1.18	1.245
420	Cdca5	1.18	0.74
421	Prmt5	1.18	0.73
422	Pycr1	1.18	1.14
423	Dck	1.173333333	0.87
424	Angptl7	1.17	0.75
425	Gm10439; Gm15080; Gm15085; Gm15093; Gm15107; Gm15109; Gm15114; Gm15128; Luzp4; Ott	1.17	0.85
426	Mars	1.17	0.79
427	Smad7	1.165	0.7
428	Psip1	1.165	0.825
429	Efnb2	1.155	0.7875
430	Tpx2	1.15	0.705
431	Gm10362; Rpl17	1.15	0.94
432	Pim1	1.15	0.66
433	Rrp9	1.15	0.78
434	Akr1b7	1.15	0.67
435	Wdr75	1.145	0.68
436	Gclc	1.145	1.25
437	Wdr36	1.145	0.855
438	Rcl1	1.14	0.74
439	Eprs	1.1375	1.075
440	Yars2	1.135	0.71
441	Phyhd1	1.13	1.34
442	Mir17hg	1.13	0.66
443	Slc30a4	1.13	0.9
444	Ccnf	1.12	0.785
445	Rassf5	1.11	1.2
446	Gtpbp2	1.11	0.7
447	Polr3g	1.11	0.98
448	Zfp317	1.11	0.95
449	Acyp2	1.11	0.83
450	Hist1h2ao; Hist1h2ap	1.11	0.66
451	Vldlr	1.105	1.145
452	Dpysl3	1.103333333	0.936666667
453	Msh6	1.1	0.74
454	Dctpp1	1.1	0.75
455	Snord22	1.1	0.73
456	Pdcd2	1.1	0.65
457	Slc19a2	1.1	0.93
458	Zfp850	1.1	1.08
459	Fbl; LOC1026432	1.095	0.825
460	Adh7	1.09	1.015

461	Akr1b3	1.09	1.13
462	Eed	1.09	0.67
463	Hat1	1.09	0.71
464	Selenbp1	1.085	1.145
465	Gfer	1.085	0.66
466	Pald1	1.08	0.8
467	Raet1a;b,c,d,e	1.08	1.22
468	Nav3	1.075	0.7
469	Slc39a10	1.075	0.74
470	Dnajc2	1.07	0.695
471	Negr1	1.07	1.2
472	Eif3d	1.07	0.85
473	LOC102641332; Ran	1.066	0.682
474	Fbxo17	1.06	0.8
475	Cdc25b	1.06	0.75
476	Snhg6	1.06	0.95
477	C1qbp	1.055	0.705
478	Zfp7	1.05	0.79
479	Arl14ep	1.046666667	0.853333333
480	P4ha2	1.04	0.83
481	Cyp4b1	1.04	1.52
482	B230311B06Rik	1.04	0.68
483	Plk1	1.03	0.9
484	Ccdc107	1.03	0.67
485	Kif18b	1.03	0.67
486	4933413G19Rik; Foxm1	1.03	0.75
487	Mcu	1.025	0.74
488	Siah2	1.02	0.66
489	Avpi1	1.02	1
490	Dars	1.02	0.7
491	Cox6a2	1.015	1.025
492	Egln3	1.015	1.015
493	Pfkp	1.013333333	1.153333333
494	Pvrl3	1.013333333	0.74
495	Cd274	1.01	0.66
496	Ska3	1.01	0.72
497	Hmga1; Hmga1-rs1	1.01	0.83
498	Inafm2	1.01	0.68
499	Ipo7	1.01	1.04
500	Pcgf5	1.01	0.73
501	Tmem209	1.01	0.78
502	Pla2g16	1	0.965

Data about *cMyc* and *N-myc* gene expression were marked with pink and green, respectively.
